# IL-1β-primed mesenchymal stromal cells exert enhanced therapeutic effects to alleviate Chronic Prostatitis/Chronic Pelvic Pain Syndrome through systemic immunity

**DOI:** 10.1186/s13287-021-02579-0

**Published:** 2021-09-25

**Authors:** Hanchao Liu, Xinning Zhu, Xiaohui Cao, Ani Chi, Jian Dai, Zhenqing Wang, Chunhua Deng, Min Zhang

**Affiliations:** 1 Department of Andrology, The First Affiliated Hospital of Sun Yat-Sen University, No. 58 Zhongshan Second Road, Guangzhou, China; 2 Reproductive Medicine Research Center, The Sixth Affiliated Hospital of Sun Yat-Sen University, Guangzhou, China; 3 Hubei Key Laboratory for Kidney Disease Pathogenesis and Intervention, School of Medicine, Hubei Polytechnic University, 16 North Guilin Road, Huangshi, 435003 Hubei China; 4 Guangdong Provincial Key Laboratory of Orthopaedics and Traumatology, The First Affiliated Hospital of Sun Yat-Sen University, Guangzhou, 51008 China

**Keywords:** IL-1β-primed MSCs, Systemic immunity, Immunoregulation, CP/CPPS, Pain

## Abstract

**Background:**

Chronic prostatitis/chronic pelvic pain syndrome (CP/CPPS) seriously affects patient health. Despite the elusiveness of innate therapeutic effects, mesenchymal stromal cells (MSCs) hold great promise for inflammation-related diseases. Recent evidence indicates that disease-specific inflammatory cytokines could enhance the therapeutic effects of MSCs.

**Methods:**

By establishing a CP/CPPS mouse model and pretreating MSCs with the cytokine interleukin-1β (IL-1β), we studied the IL-1β-primed MSC immunoregulatory ability and targeted migration ability in vitro and in CP/CPPS mice.

**Results:**

IL-1β levels significantly increased in the prostate tissue and serum of experimental autoimmune prostatitis (EAP) mice. Pretreatment with IL-1β enhanced the immunomodulatory potential and targeted migration of MSCs in vitro. Furthermore, intravenous infusion of IL-1β-primed MSCs dampened inflammation in prostate tissues and alleviated hyperalgesia in EAP mice. The infused MSCs inhibited monocyte infiltration and promoted regulatory T lymphocyte formation in prostate tissue, thus remodeling the local environment. Surprisingly, IL-1β-primed MSCs exhibited improved accumulation in the spleen but not in prostate tissue. Accordingly, infused MSCs reshaped systemic immunity by reducing the proportion of Ly6C^high^CD11b^+^ monocytes and boosting the proportion of CD4^+^Foxp3^+^ regulatory T lymphocytes in the spleen and lung. Inflammatory chemokine (C–C motif) ligand 2 (CCL2) decreased through the downregulation of the NF-κB and JNK/MAPK pathways by inflammatory resolution via MSCs infusion to alleviate pain.

**Conclusion:**

In summary, IL-1β-primed MSCs restored systemic immunologic homeostasis to alleviate CP/CPPS by modulating systemic immunity. These findings provide a novel strategy to boost the therapeutic effects of MSC-based therapy for CP/CPPS and reveal the essential role of systematic immunity in the treatment of CP/CPPS with MSC infusion.

**Supplementary Information:**

The online version contains supplementary material available at 10.1186/s13287-021-02579-0.

## Introduction

Chronic prostatitis/chronic pelvic pain syndrome (CP/CPPS) is a common disorder with chronic pelvic pain, prostatic inflammation, lower urinary tract symptoms, and sexual dysfunction [1, 2]. Epidemiologic studies have shown that CP/CPPS accounts for approximately 90–95% of all prostatitis diagnoses and affects approximately 8–11.5% of men younger than 50 years [3, 4], comparable to ischemic heart disease and diabetes mellitus. Unfortunately, chronic urological pain is closely associated with psychosocial dysfunction, including depression, anxiety, and catastrophizing, thus severely impacting patient quality of life [1]. Like other chronic pain disorders, the etiology and efficient treatment of CP/CPPS is still enigmatic [1, 3]. Recent evidence suggests that immunological disorders are pivotal in the pathophysiological progression of CP/CPPS and other chronic pain syndromes [4–7]. For example, Mailhot et al. reported that interleukin-1β (IL-1β) drives chronic pain in multiple sclerosis, arthritis, and osteoarthritis [8]. Clinical trials have demonstrated that anti-inflammatory agents, including cyclooxygenase and corticosteroids, could lead to symptomatic improvement in CP/CPPS patients [9, 10]. However, due to the nonspecific suppression of immunity as a whole, immunosuppressive agents inevitably aggravate immune disorders when administered in the long-term. Possessing the capacity to rebalance dysregulated immunity, mesenchymal stromal cells (MSCs) have been intensively investigated in various preclinical studies and clinical trials for their translational potential in combating chronic immunological disorders [11]. Therefore, it is imperative to elucidate the therapeutic effects of MSC-based therapy for CP/CPPS.

Although encouraging, the innate therapeutic outcomes of MSC-based therapies are limited and variable [12, 13]. Therefore, it is one of the major challenges to explore priming strategies specifically tailored to optimize MSC potency for specific disease indications [14]. Stem cells possess the ability to sense environmental pressures and actively alter their epigenetic landscapes and corresponding functions to rapidly adapt to secondary assaults [15]. Notably, MSCs could also trigger a memory effect after a transient stimulus [14]. For example, Li et al. demonstrated that mechanical stimulus in the culture expansion period elicited the myofibroblast memory of MSCs and was sufficient to exert biased fibrogenic differentiation after transplantation in vivo [16]. That is, a specific stimulus in vitro could poise MSCs to remember the stress and avoid the lag time for in vivo activation after infusion in specific clinical settings. It is generally accepted that pretreatment with inflammatory cytokines could enhance the immunomodulatory functions of MSCs and their potential therapeutic efficiencies by imitating the in vivo inflammatory context [11, 17]. For example, preconditioning MSCs with inflammatory cytokines was shown to increase the secretion of immunomodulatory molecules, including TNF-stimulated gene 6 protein (TSG-6), Prostaglandin E2 (PGE-2), and galectins [17]. Therefore, it is pivotal to explore appropriate priming strategies to facilitate MSC-based therapies for CP/CPPS.

The mechanisms of MSC-mediated immunomodulation are multifaceted in a disease-dependent manner [11, 17–19]. In general, MSCs can orchestrate local and systemic immunity in the context of an inflammatory environment [11, 17–19]. It is believed to be essential for the therapeutic efficiencies of MSC infusion that the abilities of exogenously delivered MSCs home to the lesion tissue to remodel the local microenvironment [20–22]. However, other researchers argue that the therapeutic functions of systemically administered MSCs rely on their abilities to restore systemic immunological homeostasis [23–25]. For example, Murphy et al. revealed that TNF-α/IL-1β-primed MSCs could sustain corneal allograft survival via the induction of regulatory T lymphocyte expansion in the lung [25]. Huang et al. found that MSCs could ameliorate spontaneous infection of acute ischemic stroke by preserving spleen B cells via mitochondrial transfer [26]. Moreover, Yi et al. demonstrated that infused MSCs hardly migrate into prostate tissues in chronic bacterial prostatitis rat models due to the blood-prostate barrier [27]. Therefore, further investigation is needed to clarify the influence of MSC infusion on systematic and local immunity in CP/CPPS.

In this study, through the establishment of a CP/CPPS mouse model, we found that the expression of IL-1β was widely increased in the spleen, bloodstream, and prostate. After priming MSCs with IL-1β, it was discovered that the NF-κB pathway of MSCs was activated. The TSG-6 and COX-2 pathways were more highly expressed in IL-1β-primed MSCs, and the immunomodulatory ability was enhanced. The expression of chemokine C-X-C motif receptor 4 (CXCR4) increased. We further proved that the accumulation of IL-1β-primed MSCs in the spleen increased. The MSC immunomodulatory ability and targeted migration to the spleen were enhanced after IL-1β priming. After MSC infusion, the migration and differentiation of mononuclear cells/macrophages in the bloodstream, spleen, lung, and prostate decreased. The number of CD4^+^Foxp3^+^ Treg cells in the bloodstream, spleen, lung, and prostate increased. Anti-inflammatory macrophages in the prostate were increased, and inflammation in the prostate was alleviated. The pain-related inflammatory cytokine chemokine (C–C motif) ligand 2 (CCL2) was reduced in the prostate through MSC immunoregulation in systemic immunity.

## Method

### Mice and cell resources

Human umbilical cord samples were collected from six full-term newborns. All samples were collected with patient consent and signed informed consent. This study was approved by the Ethics Committee of the First Affiliated Hospital of Sun Yat-sen University (approval number: SYSU-IACUC-2018-168-1).

Male NOD/ShiLtJ mice (6–8 weeks old) were purchased from Jiangsu Gempharmatech. All mice were kept under controlled temperature (24 °C ± 1 °C) and relative humidity (50–60%). The light period was 12 h, and the dark period was 12 h. All mice were allowed free access to food and water. All surgical procedures and postoperative nursing methods were approved by the Animal Care and Use Committee of Sun Yat-sen University (approval number: SYSU-IACUC-2021-000164). On a specified date, the mice were euthanized by inhaling excessive amounts of isoflurane, according to the American Veterinary Medical Association (AVMA) Guidelines for the Euthanasia of Animals: 2013 Edition.

### Isolation, culture, and pretreatment of human umbilical cord-derived MSCs

As previously described, MSCs were isolated from the umbilical cord of newborns [28]. In short, the blood vessels were carefully removed from the umbilical cord, and the remaining Wharton’s jelly was cut up and digested with 1 mg/mL collagenase II (Micropore) in Hank's balanced salt solution (HBSS, HyClone) for 30 min at 37 °C. The isolated cells were filtered through a cell filter with a mesh diameter of 70 μm to obtain a cell suspension.

The cells were washed twice with HBSS and cultured in DMEM/F12 (Gibco) supplemented with 10% fetal bovine serum (FBS, HyClone), 1% nonessential amino acids (Gibco), 2 mM GlutaMAX (Gibco) and 100 IU/mL penicillin/streptomycin (Gibco) in a humidified 5% CO_2_ water-jacketed incubator (Thermo Scientific). The culture medium was changed every 3 days. Cells were passaged every 2–3 days using 0.125% trypsin (Gibco) at a split ratio of 1:3. MSCs of the 4th passage were cocultured with 10 ng/mL IL-1β (PeproTech) for 48 h.

The lentivirus carrying green fluorescent protein (GFP) was purchased from Shanghai Genechem Co., Ltd. Transfection was carried out according to the instructions. After successful transfection, flow cytometry was used to screen GFP + cells for further expansion.

### Animal model

The construction of a non-obesity diabetes-experimental autoimmune prostatitis (NOD-EAP) mouse model was performed according to a previously published article [29, 30]. The EAP mouse model was established by mixing the male reproductive accessory gland (MAG) extract of male Wistar rats with the same volume of complete Freund's adjuvant (CFA, Sigma). After repeated suction and mixing, the NOD mice were injected with a mixture of MAG extract and CFA in four different parts, namely, the neck (0.025 mL), tail (0.050 mL), and bilateral shoulders (0.050 mL), on days 0 and 15. At 30 days, MSCs, and IL-1β-primed MSCs were injected into EAP mice through the tail vein. On the indicated days, the spleen, blood, and prostate were collected for flow cytometry analysis, cytokine measurements, and histological analysis.

### Hyperalgesia behavior testing

The test was performed 30 days after the first injection and 15 days after the treatment. According to published articles, von Frey filaments were used to test hyperalgesia in the abdomen and the plantar area of the hind paw [31, 32]. In short, five different fibers were used to test the frequency of the withdrawal response by von Frey filaments applied to the pelvis and lower abdomen with forces of 0.04, 0.16, 0.4, 1.0, and 4.0 g. There are four types of behaviors that are considered to be a positive response to filament stimulation: sharp retraction of the abdomen, immediate licking, scratching of the filament stimulation area or jumping. The frequency of response was calculated as the percentage of positive responses, and the data are reported as the average percentage of the response frequency. The 50% threshold was evaluated using an up and down method [33].

### Coculture of macrophages and MSCs

The bone marrow cells surgically obtained from the femur or tibia were separated by density gradient centrifugation. The cells were grown and maintained with DMEM/F12 (HyClone) containing 10% FBS and 20% L929 conditioned medium in low adhesion plates. After 7 days of culturing, the cells were separated with TrypLETM Express (Gibco) and seeded at a density of 5 × 105 cells/well. Then, 1 μg/mL lipopolysaccharide (LPS, Sigma) was added to activate macrophages. Macrophages and saline/MSCs/IL-1β-primed MSCs were cocultured at a ratio of 5:1. After 72 h of cocultivation, the cells were collected and analyzed by flow cytometry.

### Coculture of CD4^+^ cells and MSCs

T lymphocytes were obtained from the spleen using the BioLegend Natural CD4 + T Cell Purification Kit (BioLegend) and then seeded on anti-CD3-coated plates at a density of 5 × 105 cells/well. CD4 + T cells and saline/MSCs/IL-1β-primed MSCs were cocultured at a ratio of 5:1. Treg cell differentiation was induced with RPMI 1640 containing 10% FBS, 3 μg/mL anti-CD28 (BioLegend), 50 ng/mL IL-2 (Novoprotein), and 5 ng/mL TGF-β (Novoprotein). After 72 h of cocultivation, CD4 + FOXP3 + Treg cells were collected and analyzed by flow cytometry.

### Ca^2+^ imaging in DRGs

According to the previously published literature [34, 35], dorsal root ganglia (DRGs) (T13-L2) were isolated from male mice aged 4 to 6 weeks and incubated with collagenase (Sigma). They were then incubated at 37 °C for 45 min, digested with 0.05% trypsin–EDTA (Gibco), resuspended in complete medium (DMEM/F12), and cultured on coverslips for 3 days. A normal physiological salt wash (containing 140 mM NaCl, 5 mM KCl, 1 mM MgCl_2_, 2.5 mM CaCl_2_, 10 mM HEPES, and 10 mM glucose) was used for washing 3 times, and the pH was adjusted to 7.4. The cells were incubated with 5 μM fluo 3-AM (Molecular Probes) for 60 min at room temperature. The fluorescence signal was recorded by a laser scanning imaging system (IX83 research inverted microscope, Japan). Fluo 3 is excited at 488 nm and analyzed at 530 nm. After equilibrating for 3–5 min, the cells were treated with CCL2 and capsaicin to increase Ca^2+^ influx. The Ca^2+^ signal was captured in a 4 s interval.

### Transwell migration assay

An 8-μm pore membrane filter (Millipore) was used to evaluate the targeted migration ability of MSCs. Serum-free starved MSCs, and IL-1β-primed MSCs were inoculated in the upper chamber (1 × 10^5^ cells per well), while the lower chamber was filled with 500 μL of DMEM/F12, which contained 50 ng/mL mouse Chemokine (C-X-Cmotif) ligand 12 (CXCL12, PeproTech). After incubating in 5% CO_2_ at 37 °C for 12 h, the upper chamber of the filter was removed. After fixing with 4% paraformaldehyde (PFA), the cells that migrated to the lower chambers were stained with 0.1% crystal violet and then counted by microscopy.

### Flow cytometry

The obtained tissues were cut into small pieces and shaken with Liberase TL (100 U/mL; Roche) with DNase I (100 U/mL; Sigma) in a 37 °C water bath for 20 min. Subsequently, the cell suspensions were passed through a 70-μm cell strainer to produce single cells. The collected peripheral blood samples were subjected to red blood cell lysis (BioLegend) and then washed twice with PBS. Single-cell suspensions, tissues, and blood samples were incubated with the appropriate antibodies (Additional file 1: Table S1) for 15 min in the dark. The cells were washed twice, resuspended in PBS, and analyzed by MoFlo Astrios EQ (Beckman Coulter) for at least three independent experiments. The data were analyzed using FlowJo V10.0 (Tree Star). Information on the antibodies is listed in Additional file 1: Table S1.

### Quantitative real-time PCR (RT-qPCR)

Total RNA was extracted and purified by NucleoZol reagent (Macherey–Nagel), and cDNA was obtained by a cDNA library kit (Takara). qRT-PCR was conducted on a Bio-Rad CFX96TM detection system (Roche) with SYBR PCR Master Mix (Roche). β-Actin was used for standardization. The primers used are listed in Additional file 1: Table S2.

### Tissue preparations and histological analysis

Tissue was embedded in a tissue-Tek optimum cutting temperature (OCT) complex and frozen in a cryostat (Leica CM1900, Germany). Sections of 5-μm thickness were then placed on glass slides (Bio-Optical). Slices were prepared and sealed with 0.05% thiobarbital sodium-Tween containing 0.5% FBS for 30 min, incubated overnight with the primary antibody or an IgG control antibody at 4 ℃, and incubated for 60 min with the secondary antibody at room temperature. The slices were re-stained with 4',6-diamidino-2-phenylindole (DAPI) for 3 min at room temperature. Immunofluorescence (IF) signals were visualized and recorded using a laser scanning confocal microscope (LSM780, Zeiss, Germany). For hematoxylin–eosin (HE) staining, the tissue was fixed overnight in 4% PFA (Sigma), continuously dehydrated and embedded in paraffin. Then, slices with a thickness of 5 μm were placed on glass slides (Bio-Optical). The cells were stained with an HE staining kit (Servicebio). All the primary and secondary antibodies are listed in Additional file 1: Table S2.

### Western blot analysis

Cultured cells or tissues were homogenized with RIPA lysis buffer (Millipore) containing protease inhibitors for over 30 min. Nuclear protein lysates were generated using a nuclear protein extract kit (Servicebio) following the manufacturer’s instructions. The homogenates were centrifuged at 15,000 × g at 4 °C for 10 min and then the protein concentration was detected by a BCA protein analysis kit (Sigma). Approximately 20–40 μg of total protein solution was loaded onto SDS–polyacrylamide gels and transferred onto 0.22-μm polyvinylidene fluoride (PVDF) membranes (Millipore). The membranes were blocked with 5% bovine serum albumin for 30 min at room temperature and then probed with the indicated primary antibody at 4 °C overnight, followed by incubation with the horseradish peroxidase-conjugated secondary antibody at room temperature for 1 h. A chemiluminescence kit (Millipore) was used to detect the target bands. Information about the antibodies used is provided in Additional file 1: Table S1.

### Cytokine measurements

Tissue was homogenized with TissueLyser II (QIAGEN) and 5-mm steel balls (QIAGEN) in tissue protein extraction reagent (Thermo Fisher), and the supernatant was obtained after centrifugation. Blood samples were collected, and serum was extracted by centrifugation. According to the manufacturer's instructions, the LEGENDplex™ mouse inflammatory panel (BioLegend) was used to detect the concentration of 13 cytokines in tissue culture supernatants, serum samples and lysates extracted from RIPA. Vacuum freeze-drying equipment (Songyuan) was used to concentrate the extract to obtain the appropriate concentration. A BCA protein analysis kit (Sigma) was used to determine the concentration of total protein in the lysate extracted by RIPA, and the same amount of total protein was used for subsequent detection. The kit provides capture beads that bind to specific antibodies, making them easily distinguishable by size and fluorescence signal. The biological sample was incubated with the capture beads at room temperature for 2 h and then a biotin-labeled detection antibody was added for flow cytometry detection. LEGENDplex 8.0 data analysis software was used to calculate the average fluorescence intensity corresponding to each cytokine. The cytokine concentration was measured by comparing the fluorescence intensity with a standard value.

### Statistics

Comparisons between groups were performed by t-test or one-way analysis of variance (ANOVA), and Tukey's multiple comparison test was used. Error bars indicate the standard error of the mean. *P* < 0.05 was considered statistically significant. All statistical analyses were performed with GraphPad Prism 8.0 software.

## Result

### IL-1β is elevated in the spleen, bloodstream, and prostate of NOD-EAP mice.

**Fig. 1:**
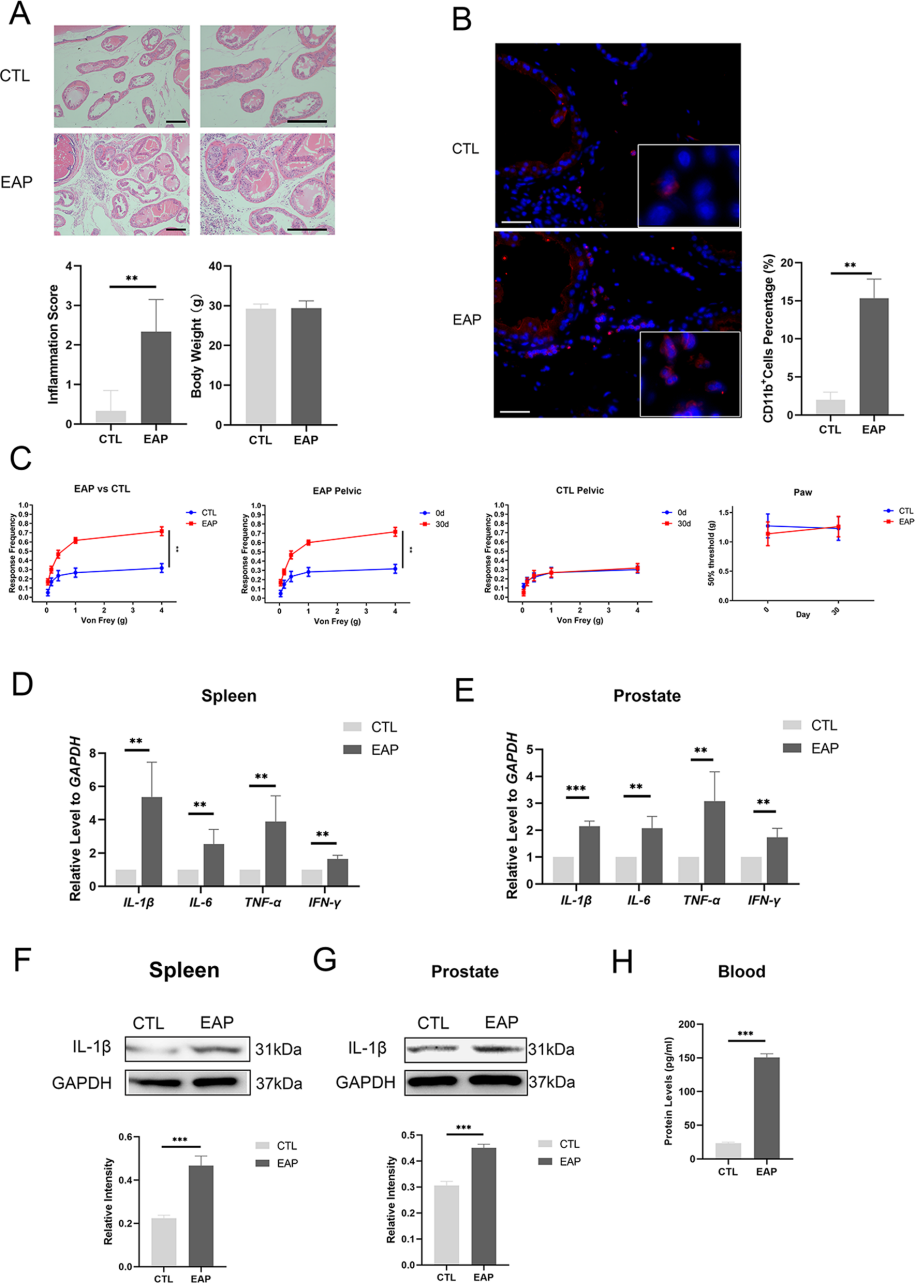
IL-1β is widely elevated in the spleen, prostate, and bloodstream of NOD-EAP mice. **A** HE staining showed inflammation and the structure of the prostate in EAP mice and control mice (bar = 200 μm). Inflammation score and weight in the control group and EAP group. **B** CD11b^+^ cells of the prostate in EAP mice and control mice (bar = 50 μm). **C** Hyperalgesia test to detect pelvic pain in EAP mice by von Frey filaments (*n* = 6 per group). **D** IL-1β mRNA expression in the spleen of EAP mice and control mice by RT-qPCR (*n* = 6 per group). **E** IL-1β mRNA expression in the prostate of CP/CPPS mice and control mice by RT-qPCR. **F** IL-1β protein expression in the spleen of CP/CPPS mice and control mice by Western blotting (*n* = 6 per group). **G** IL-1β protein expression in the prostate of CP/CPPS mice and control mice by Western blotting. **H** IL-1β protein expression in the bloodstream of CP/CPPS mice and control mice by Legendplex™ bead-based immunoassays (*n* = 6 per group). Error bars indicate SD. *p < 0.05, **p < 0.01, ***p < 0.001

The etiology, pathogenesis, and optimal treatment of CP/CPPS are poorly understood, and we have adopted the most recognized and applied CP/CPPS animal model (NOD-EAP mouse model) by CP/CPPS researchers [36, 37]. HE staining and pain behavior detection proved that the NOD-EAP mouse model was successfully established. At 30th day after the 1st immunization, prostate HE staining in the CP/CPPS mice showed a large amount of inflammatory cell infiltration and tissue structure destruction (Fig. 1A). The inflammation scores significantly increased in the model group compared to the control group, meanwhile the body mass between the two groups has no significant difference (Fig. 1A). Moreover, CD11b^+^cells were significantly increased in the prostate of EAP group, which suggesting that the inflammation state in EAP mice was aggravated (Fig. 1B). The pelvic pain between the control group and the model group were significantly different, indicating that the mice in the model group had significant pelvic pain (Fig. 1C). The mice in the control group showed no significant differences in pelvic pain at 0 and 30 days (Fig. 1C). The behavior detection of paw pain showed no significant differences, which indicates that this pain is pelvic-specific pain (Fig. 1C). The pelvic pain difference between 0 and 30 days was statistically significant in EAP mice (Fig. 1C).

We have mentioned that the immunomodulatory ability and MSC homing efficiency of MSCs can be enhanced by cytokines [38–40]. As an inflammatory cytokine, IL-1β plays a critical role in autoimmune conditions, inflammation and neuropathic pain [41]. IL-1β was widely expressed in the spleen and prostate of CP/CPPS mice by RT-qPCR and Western blotting. The mRNA expression levels of inflammatory factors were significantly different in the spleens (Fig. 1D) and prostates (Fig. 1E) of the model group and the control group (Fig. 1D). Western blotting showed that the expression levels of IL-1β in the spleen (Fig. 1F) and prostate (Fig. 1G) were significantly increased in the model group. Moreover, the Biolegendplex™ panel showed that serum IL-1β was significantly increased in the model group (Fig. 1H). As a highly expressed inflammatory cytokine in the spleen, bloodstream, and prostate of CP/CPPS mice, IL-1β was our ideal pretreatment factor.

### IL-1β-primed MSCs showed better immune regulation ability and targeted migration ability than MSCs in vitro

**Fig. 2:**
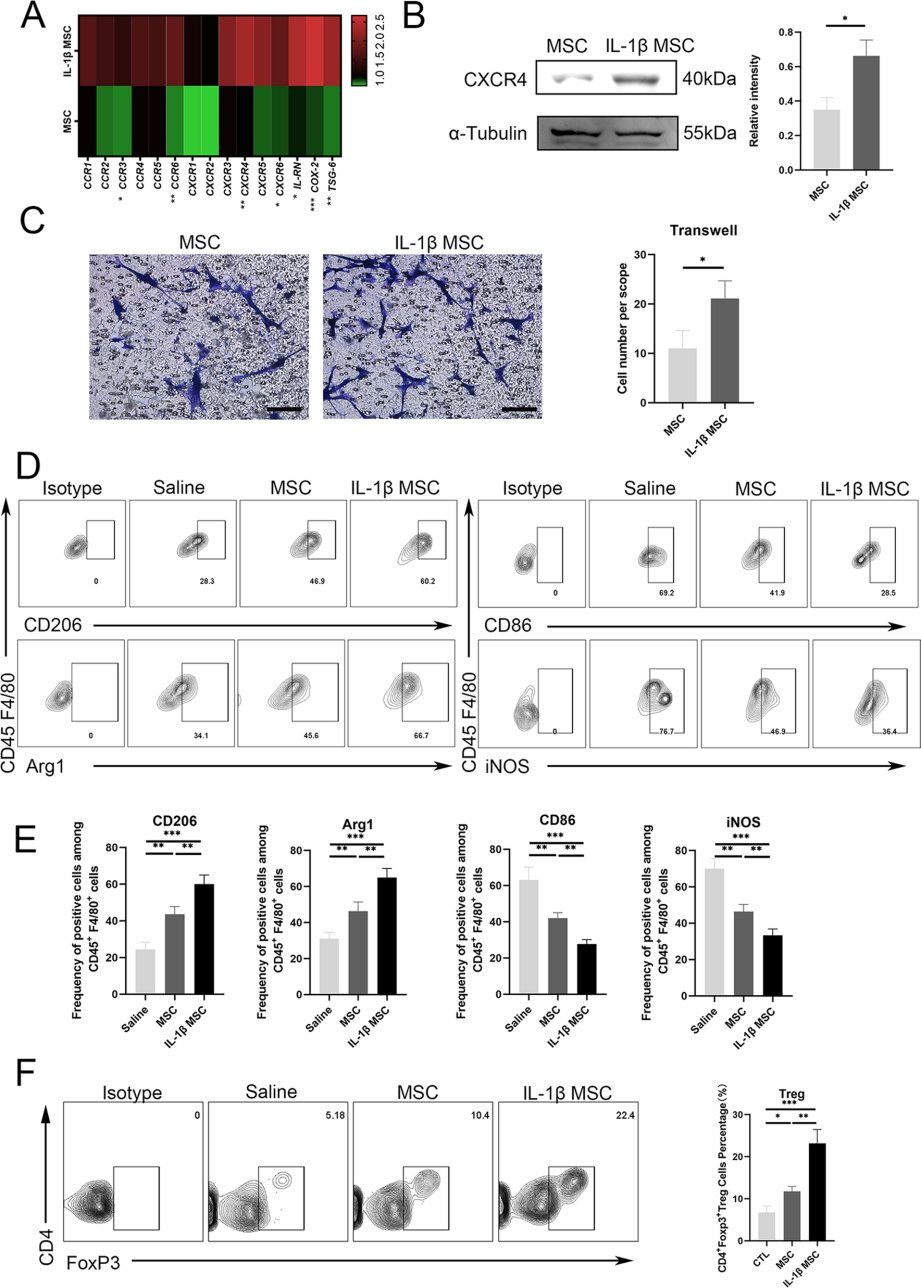
IL-1β-primed MSCs showed better immune regulation ability and targeted migration ability than MSCs in vitro. **A** The expression of CXCR4, COX-2, TSG-6 and some other genes was checked by RT-qPCR in MSCs, and IL-1β-primed MSCs (*n* = 3 per group). **B** IL-1β-primed MSCs and MSCs expressing the CXCR4 protein were assessed by Western blotting (*n* = 3 per group). **C** The migration ability of IL-1β-primed MSCs and MSCs targeting CXCL12 was assessed by Transwell assays (*n* = 3 per group) (bar = 100 μm). **D** Inflammatory properties: Representative flow cytometry histograms of macrophages with CD206, Arg1, CD86, and iNOS in the saline, MSC, and IL-1β-primed MSC groups were detected by flow cytometry (*n* = 3 per group). **E** Statistical histograms of macrophages with CD206^+^, Arg1^+^, CD86^+^, and iNOS^+^ quantitative analysis in the saline, MSC, and IL-1β-primed MSC groups. **F** The proportion of CD4^+^FoxP3^+^ Treg cells in the saline, MSC, and IL-1β-primed MSC groups were detected by flow cytometry (*n* = 3 per group). Error bars indicate SD. *p < 0.05, **p < 0.01, ***p < 0.001

Recently, researchers have discovered the enhanced immunoregulatory ability and MSC homing efficiency of cytokine-primed MSCs due to the activation of the NF-κB pathway [42, 43]. IL-1β activated the NF-κB pathway of MSCs via phosphorylation of P65, as shown by Western blot analysis (Additional file 2: Figure S1A). Flow cytometry analysis showed that IL-1β did not alter the expression patterns of MSC-specific surface markers. The proliferation ability and appearance were not significantly different between the two groups (Additional file 2: Figure S1B, C, D). Activation of NF-κB in MSCs after IL-1β priming led to the increased secretion of downstream immunomodulatory proteins and chemokine receptors [44, 45]. IL-1β-primed MSCs showed the activation of CXCR4 and COX-2, TSG-6 and some other genes by RT-qPCR (Fig. 2A). Activation of the CXCL12/CXCR4 axis promoted the MSC secretion of pro-survival and pro-angiogenic cytokines and the targeted engraftment ability of MSCs [46, 47]. The upregulation of the chemokine receptor CXCR4 proved that its migratory ability in response to CXCL12 was enhanced. Western blotting verified that CXCR4 expression in primed MSCs increased (Fig. 2B). In the Transwell experiment, CXCL12 protein was placed in the lower chamber of the Transwell, and the IL-1β-primed MSC-targeted migration ability in response to CXCL12 was enhanced (Fig. 2C). The scratch experiment proved that the migration ability of MSCs after priming was also enhanced (Additional file 2: Figure S1E). In vitro, MSCs and mouse bone marrow-derived macrophages were cocultured, and it was found that IL-1β-primed MSCs regulated the anti-inflammatory properties of CD45 + F4/80 + macrophages with increased CD206^+^ or arginase 1 (Arg1^+^) frequency and regulated the proinflammatory properties of CD45 + F4/80 + macrophages with decreased CD86^+^ or inducible nitric oxide synthase enzyme (iNOS^+^) frequency (Fig. 2D, E). Gate selection is presented (Additional file 2: Figure S2A). To immunoregulate the proportion of Treg cells, MSCs and mouse spleen CD4^+^ T cells were cocultured, and it was found that IL-1β-primed MSCs maintained a higher proportion of Treg cells (Fig. 2F); gate selection was presented (Additional file 2: Figure S2B). In vitro, we confirmed that the immunoregulatory and target migration abilities were enhanced in IL-1β-primed MSCs.

### IL-1β-primed MSCs targeted migration to the spleen more than MSCs

**Fig. 3:**
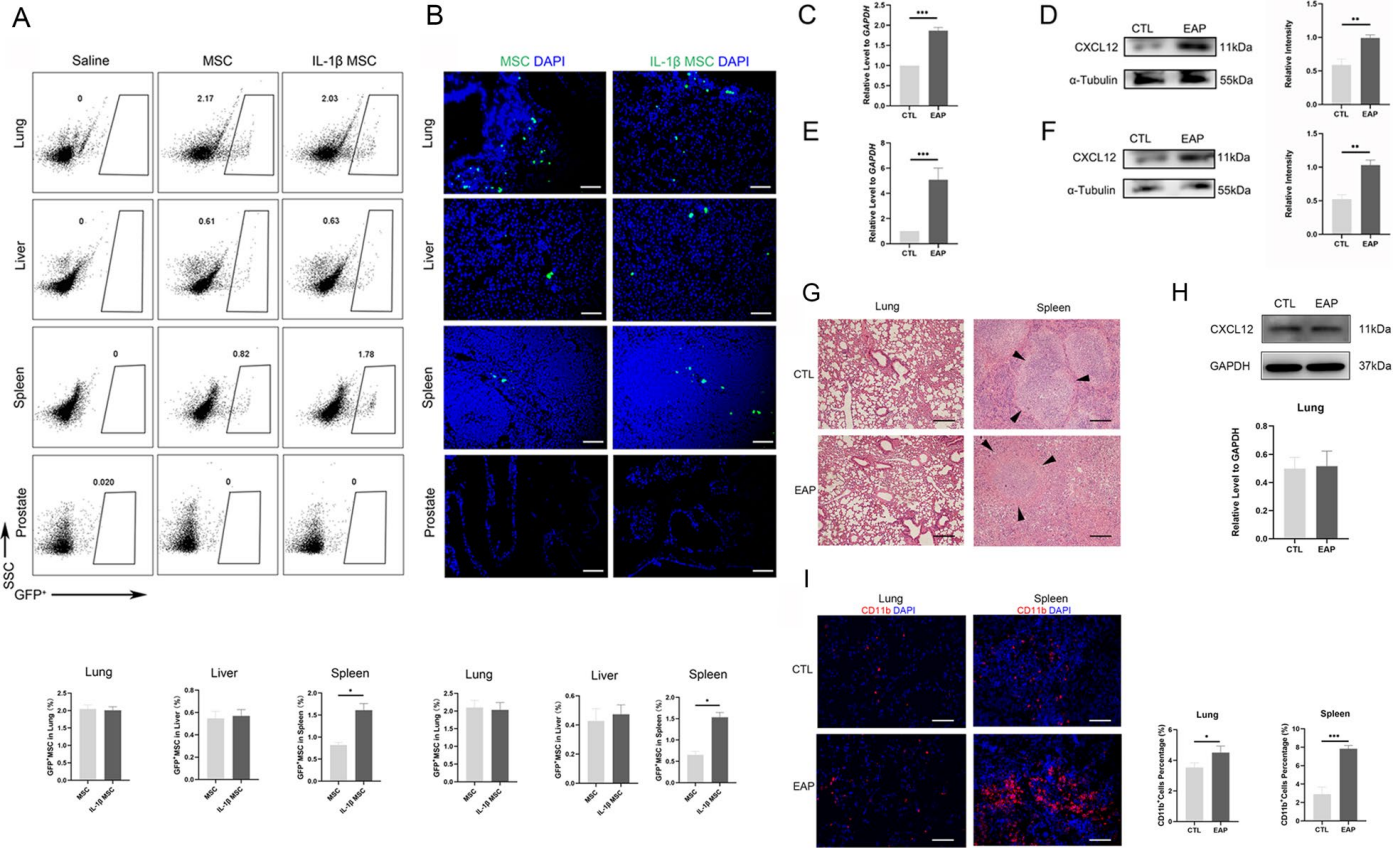
IL-1β-primed MSCs migrated to the spleen more than MSCs. **A** GFP^+^ IL-1β-primed MSCs and GFP^+^ MSCs were recruited to the lungs, livers, spleens, and prostates of EAP mice by flow cytometry (*n* = 6 per group). **B** GFP^+^ IL-1β-primed MSCs and GFP^+^ MSCs were recruited to the lungs, livers, spleens, and prostates of EAP mice by IF (bar = 100 μm) (*n* = 6 per group). **C** CXCL12 mRNA expression in the spleens of EAP mice and control mice by RT-qPCR (*n* = 6 per group). **D** CXCL12 protein expression in the spleens of EAP mice and control mice by Western blotting (*n* = 6 per group). **E** CXCL12 mRNA expression in the prostates of EAP mice and control mice by RT-qPCR (*n* = 6 per group). **F** CXCL12 protein expression in the prostates of EAP mice and control mice by Western blotting (*n* = 6 per group). **G** HE changes of lung and spleen in control and EAP mice (Bar = 100 μm). **H** The CXCL12 expression of lung in control and EAP mice by Western blot (*n* = 6 per group). **I** CD11b^+^ immune cells in lung and spleen in control and EAP mice (Bar = 50 μm). Error bars indicate SD. *p < 0.05, **p < 0.01, ***p < 0.001

MSC homing and engraftment efficiency can be enhanced by inflammatory cytokines. However, the existence of a physiological barrier, such as the blood–brain barrier and blood-testis barrier, allowing MSCs to home to the lesion by intravenous infusion is still limited and controversial [48]. Certainly, MSCs not only exert regenerative effects on the local environment but also reshape the systemic immune response to facilitate inflammation resolution via systemic immune cells [23, 49]. Viruses with the GFP^+^ gene were transfected into MSCs to obtain GFP^+^ MSCs. Then, GFP^+^ MSCs were primed by IL-1β (Additional file 2: Figure S3A, B). The GFP^+^ MSCs were injected into CP/CPPS mice. We obtained the lung, liver, spleen, and prostate on the first day after the injection of GFP^+^ MSCs and GFP^+^ IL-1β-primed MSCs. GFP^+^ MSCs and GFP^+^ IL-1β-primed MSCs did not migrate to the prostate, as determined by flow cytometry and IF. Flow cytometry showed that GFP^+^ IL-1β-primed MSCs engrafted in the spleen were more abundant than GFP^+^ MSCs, but this was not found in the prostate. There were no significant differences in MSC and IL-1β MSC engraftment into the lung or liver (Fig. 3A). IF showed that GFP^+^ IL-1β-primed MSCs engrafted in the spleen were more abundant than GFP^+^ MSCs and were not in the prostate. There were no significant differences in MSC and IL-1β MSC engraftment into the lung or liver (Fig. 3B). After the pretreatment of MSCs with IL-1β, the expression of CXCR4 in MSCs increased, and we detected the protein CXCR4 gamete CXCL12 in the mouse spleen and prostate. It was found that CXCL12 was elevated in CP/CPPS mice. CXCL12 mRNA expression increased in the spleen of NOD-EAP mice (Fig. 3C). Western blotting showed increased expression of CXCL12 in the spleen of CP/CPPS mice (Fig. 3D). CXCL12 mRNA expression increased in the prostate of CP/CPPS mice (Fig. 3E). Western blotting showed increased expression of CXCL12 in the prostate of CP/CPPS mice (Fig. 3F; scale bar: 50 μm). As the blood-prostate barrier may impeding the engraftment of MSCs into the prostate, intravenous infused MSCs might exert therapeutic effects through systemic immunity.

We investigate the histology of the lungs and spleens by the HE staining. No surprisingly, we found that histological structure of the spleen has some changes by chronic inflammation (Micro changes in the structure of the spleen lymph nodes) (Fig. 3G). However, the lung may not be significantly affected by chronic inflammation in HE staining. Subsequently, Western blot analysis also shows that the CXCL12 expression in the spleen of EAP mice significantly increases compared to the control (Fig. 3D). Interestingly, the CXCL12 expression of the lung in control and EAP mice was no significant difference (Fig. 3H). Moreover, we employed immunofluorescence staining to investigate the expression of CD11b^+^ immune cells in the lungs and spleen. Immunofluorescence staining analysis identified that the number of CD11b^+^ cells in both the lung and spleen of EAP mice significantly increases compared to the control (Fig. 3I). This may be the reason why the migration of MSCs to the lung with or without priming was the same.

### After treatment with IL-1β-primed MSCs, proinflammatory immune monocytes/macrophages decreased and anti-inflammatory immune Treg cells increased in the spleen, lung, bloodstream and prostate

**Fig. 4:**
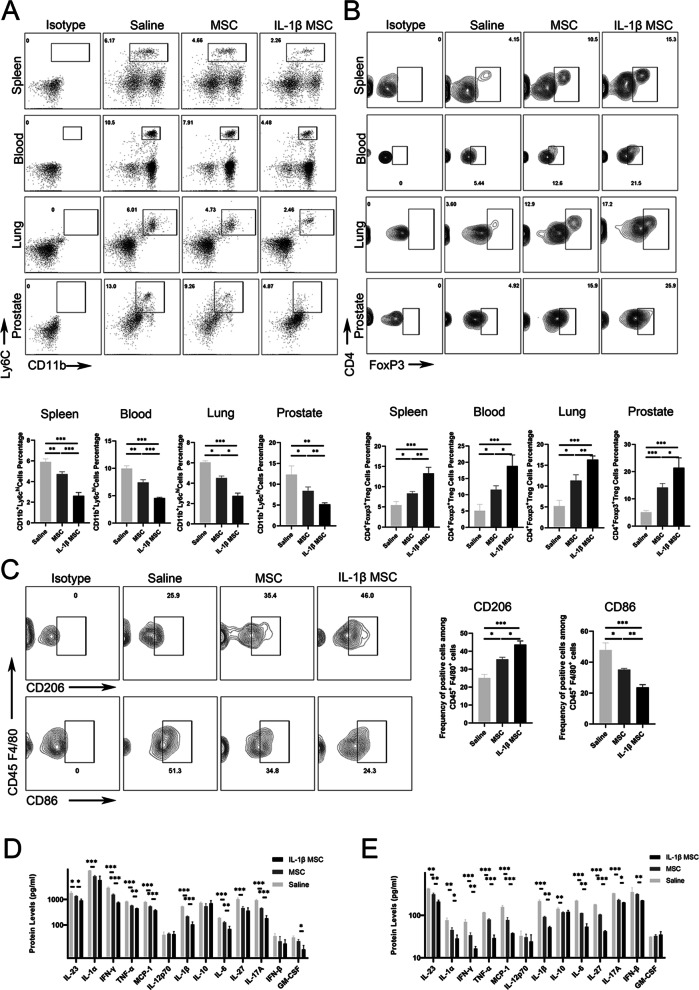
Proinflammatory immune monocytes/macrophages decreased and anti-inflammatory Treg cells increased in the spleen, bloodstream, lung, and prostate after injection of MSCs and IL-1β-primed MSCs. **A** Proportion of CD11b^+^ Ly6C^high^ cells in the spleens, bloodstreams, lungs, and prostates of the saline, MSC, and IL-1β-primed MSC groups by flow cytometry (*n* = 6 per group). **B** The proportion of CD4^+^Foxp3^+^ Treg cells in the spleens, bloodstreams, lungs, and prostates of the saline, MSC, and IL-1β-primed MSC groups (*n* = 6 per group). **C** The proportion of CD206^+^ CD45^+^F4/80^+^ macrophages in the lungs of the saline, MSC, and IL-1β-primed MSC groups, as determined by flow cytometry. The proportion of CD86^+^ CD45^+^F4/80^+^ macrophages in the lungs of the saline, MSC, and IL-1β-primed MSC groups, as determined by flow cytometry (*n* = 6 per group). **D** Inflammatory factor protein expression in the spleens of the saline, MSC, and IL-1β-primed MSC groups by Biolegendplex™ bead-based immunoassays (*n* = 6 per group). **E** Expression of inflammatory factors in the bloodstreams of the saline, MSC, and IL-1β-primed MSC groups by Biolegendplex™ bead-based immunoassays (*n* = 6 per group). Error bars indicate SD. *p < 0.05, **p < 0.01, ***p < 0.001

**Fig. 5:**
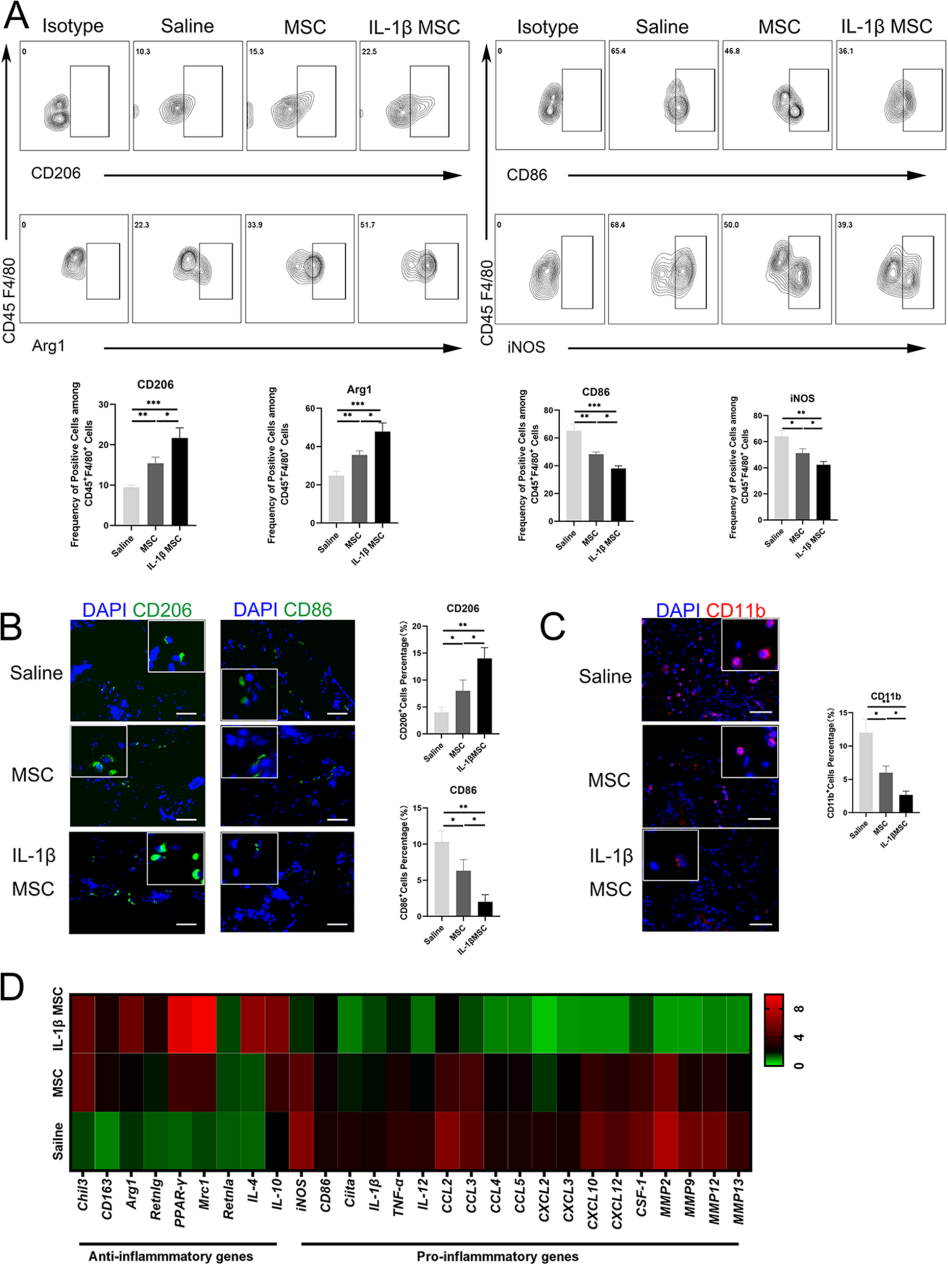
Macrophages changed from a proinflammatory phenotype to an anti-inflammatory phenotype in the prostate. **A** Proportion of CD206 and Arg1 in CD45 + F4/80 + macrophages in the prostates of the saline, MSC, and IL-1β-primed MSC groups by flow cytometry. Proportion of CD86 and iNOS in CD45 + F4/80 + macrophages in the prostates of the saline, MSC, and IL-1β-primed MSC groups determined by flow cytometry (*n* = 6 per group). **B** Proportion of CD206^+^ cells and CD86^+^ cells in the prostates of the saline, MSC, and IL-1β-primed MSC groups by IF (*n* = 6 per group, bar = 50 μm). **C** Proportion of CD11b^+^ cells in the MSC and IL-1β-primed MSC groups by IF (*n* = 6 per group, bar = 50 μm). **D** The expression of anti-inflammatory genes in in situ macrophages in the saline, MSC, and IL-1β-primed MSC groups, as determined by RT-qPCR; the expression of proinflammatory genes in in situ macrophages in the saline, MSC, and IL-1β-primed MSC groups, as determined by RT-qPCR (*n* = 6 per group). Error bars indicate SD. *p < 0.05, **p < 0.01, ***p < 0.001

Tissue monocytes are derived from CD11b^+^ monocytes in the spleen and blood [50, 51]. CD11b^+^Ly6C^high^ monocytes are inflammatory and play a critical role in the maintenance of inflammation [52]. In this study, the expression of CD11b^+^ Ly6C^high^ cells in the three groups of mice gradually decreased. The expression of Treg cells in the three groups of mice gradually increased. At the same time, the 13 inflammatory factors in the spleen and blood of mice showed that the inflammation in the saline, MSC, and IL-1β-primed MSC groups gradually decreased. The quantities of CD11b^+^ Ly6C^high^ cells gradually decreased in the spleens, blood, lungs, and prostates (Fig. 4A) of the saline, MSC, and IL-1β-primed MSC groups. In addition, Treg cells are critical for resolving inflammation and secreting regenerative cytokines [53]. The quantities of CD4^+^Foxp3^+^ Treg cells gradually increased in the spleens, blood, lungs and prostates (Fig. 4B) of the saline, MSC, and IL-1β-primed MSC groups. Flow cytometry showed that the frequency of CD206^+^ in CD45^+^CD4/80^+^ macrophages gradually increased in the prostates of the MSC and IL-1β-primed MSC groups. The frequency of CD86^+^ CD45 + CD4/80 + macrophages gradually decreased in the lungs of the MSC and IL-1β-primed MSC groups (Fig. 5C). With three different treatments, the inflammatory factors gradually decreased in the spleens (Fig. 4D) and bloodstreams (Fig. 4E) of the saline, MSC, and IL-1β-primed MSC groups, as shown by the Biolegendplex™ inflammatory panel. Gate selection was presented (Additional file 2: Figure S4). These proved MSCs have reshaped the systemic immune cells.

### Macrophage inflammatory properties changed from a proinflammatory phenotype to an anti-inflammatory phenotype in the prostate

Macrophages play a critical role in inflammation resolution and tissue healing [54, 55]. The phenotype of prostate macrophages was changed via IL-1β-primed MSC infusion. The expression of anti-inflammatory genes in in situ prostate macrophages increased after treatment, and the expression of proinflammatory genes decreased. The decreased quantities of proinflammatory macrophages in the prostate certified inflammation resolution via changes in the inflammatory properties of macrophages [54]. Flow cytometry showed that the frequencies of CD206^+^ and Arg1^+^ in CD45 + CD4/80 + macrophages gradually increased in the prostates of the MSC and IL-1β-primed MSC groups. In addition, the frequencies of CD86^+^ and iNOS^+^ in CD45^+^CD4/80^+^ macrophages gradually decreased in the prostates of the MSC and IL-1β-primed MSC group (Fig. 5A). IF showed that the expression of CD206^+^ macrophages gradually increased in the prostates of the MSC and IL-1β-primed MSC groups (Fig. 5B). IF showed that the frequency of CD86^+^ macrophages gradually decreased in the prostates of the MSC and IL-1β-primed MSC groups (Fig. 5B). IF also showed that the frequency of CD11b^+^ monocytes gradually decreased in the prostates of the MSC and IL-1β-primed MSC groups (Fig. 5C). In situ macrophages were extracted for RT-qPCR detection, and it was found that the expression of anti-inflammatory genes gradually increased in the MSC and IL-1β-primed MSC groups (Fig. 5D). Macrophage gate selection was presented (Additional file 2: Figure S5). The expression of proinflammatory genes gradually decreased in the saline, MSC, and IL-1β-primed MSC groups (Fig. 5D). The above findings might prove that although it is difficult to target MSCs to migrate to the prostate, MSCs can engraft to the spleen. IL-1β-primed MSCs regulated inflammation resolution in the prostate via systemic immune cells.

### The expression of inflammation-related pathways decreased after treatment, and inflammation resolution was promoted

**Fig. 6:**
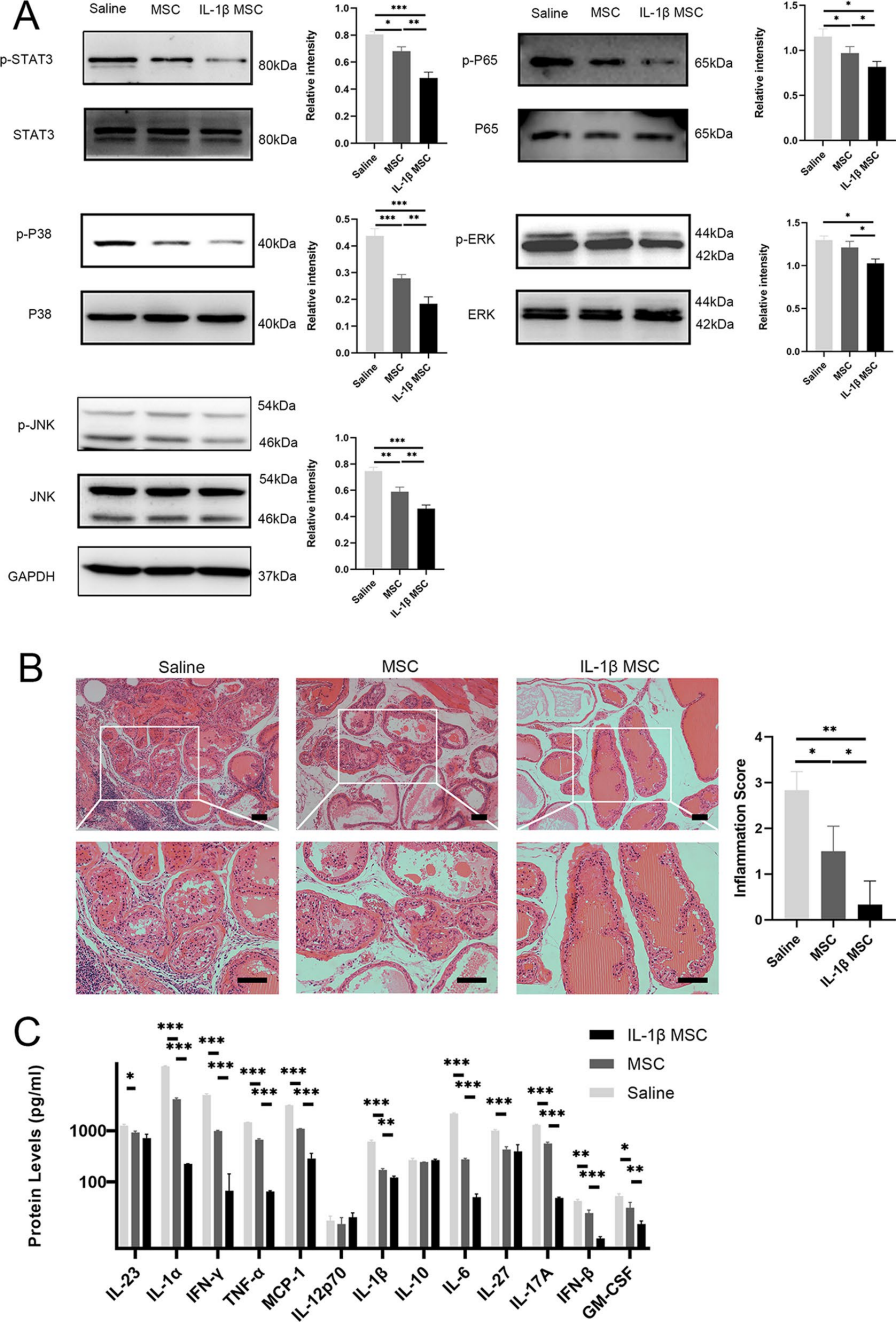
Expression of inflammation-related pathways decreased after treatment, and inflammation in the prostate was reduced. **A** Western blotting was used to detect the phosphorylation of P65, STAT3, JNK, ERK and P38 in the prostates of the saline, MSC, and IL-1β-primed MSC groups after treatment (*n* = 6 per group). **B** The inflammation score and HE-stained slices of prostate tissue indicated the inflammation level after MSC infusion (*n* = 6 per group). **C** Inflammatory factors in the prostates of the saline, MSC, and IL-1β-primed MSC groups by Biolegendplex™ bead-based immunoassays (*n* = 6 per group). Error bars indicate SD. *p < 0.05, **p < 0.01, ***p < 0.001

The NF-κB, signal transducer and activator of transcription 3 (STAT3) and MAPK pathways are very important in inflammation. The overexpression of these pathways can exacerbate and maintain inflammation [56, 57] and can be verified by detecting phosphorylated proteins. This study found that the inflammatory pathways NF-κB, STAT3 and MAPK were significantly downregulated via MSC infusion. Moreover, HE staining and inflammatory factor detection showed that inflammation was significantly decreased via MSC infusion. Western blot analysis demonstrated that p-STAT3, p-P65, p-JNK, p-ERK, and p-P38 were downregulated in the MSC-infused group (Fig. 6A). High expression of JNK/MAPK and NF-κB can promote the expression of inflammatory factors and the pain-related chemokine CCL2. The inflammation score of prostate tissue indicated that inflammatory state was reduced after treatment. After treatment, the infiltration of immune cells in the tissue was reduced, and the tissue fibrosis was reduced (Fig. 6B). The Biolegendplex™ inflammatory panel indicated that inflammation in the prostate gradually decreased after treatment (Fig. 6C). The experimental protocol is presented in Additional file 2: Figure S6.

### Pain-related indicators were relieved after treatment

**Fig. 7:**
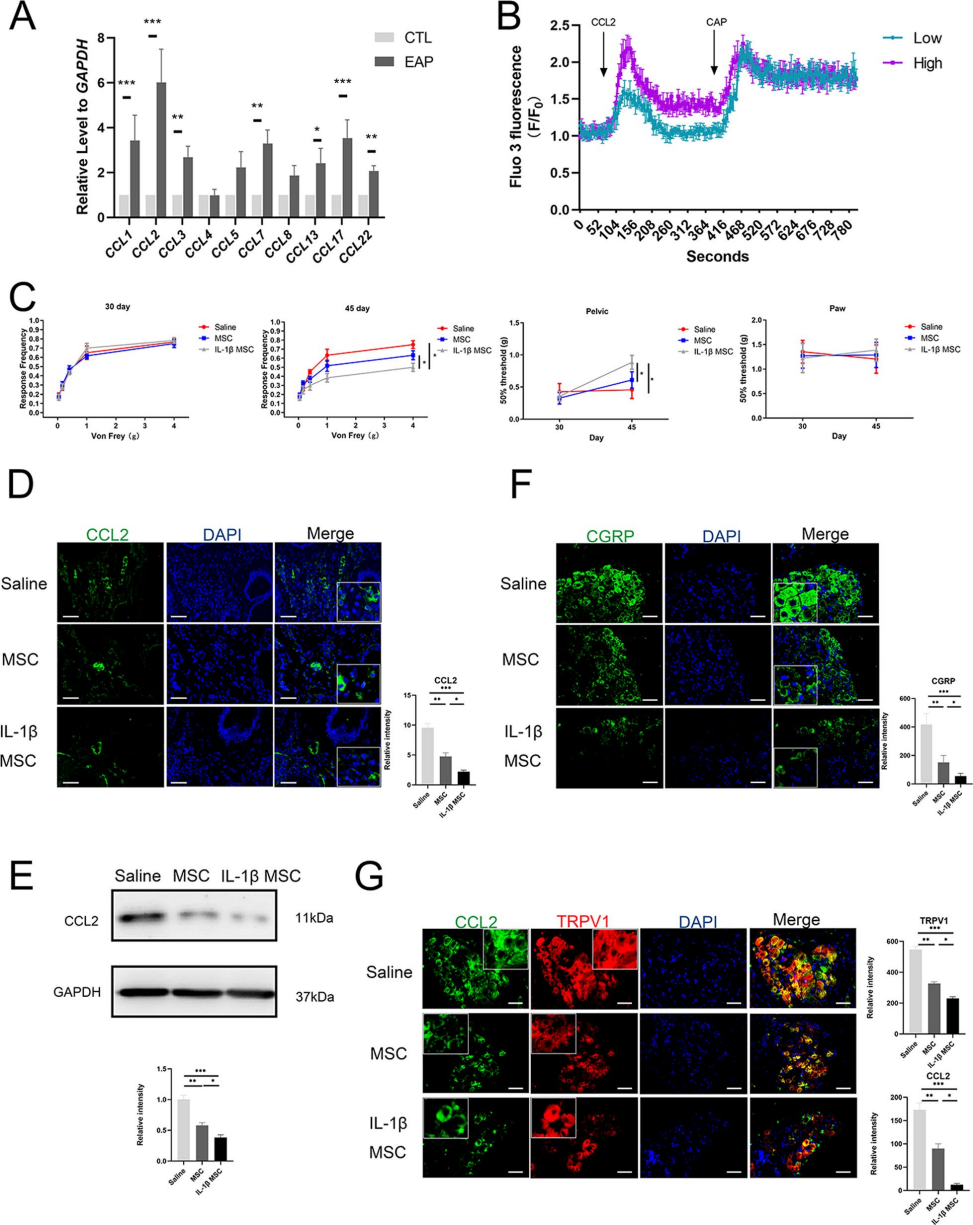
CP/CPPS pain symptoms were relieved after treatment. **A** Expression of CCL2 mRNA in EAP mice and control group mice. **B** Ca^2+^ influx in DRGs following treatment with different concentrations of CCL2. Low: 100 ng/mL; high: 300 ng/mL (*n* = 3 per group). CAP, capsaicin (300 nM). **C** At 30 days, the pelvic pain response of mice in the saline, MSC, and IL-1β-primed MSC groups; at 45 days, the pelvic pain response of mice in the saline, MSC, and IL-1β-primed MSC groups; at 30 and 45 days, the paw pain of mice in the saline, MSC, and IL-1β-primed MSC groups (*n* = 6 per group). **D** Expression of CCL2 in the prostates of the saline, MSC, and IL-1β-primed MSC groups by IF (*n* = 6 per group, bar = 50 μm). **E** Expression of CCL2 in the prostates of the saline, MSC, and IL-1β-primed MSC groups by Western blotting (*n* = 6 per group). **F** Expression of CGRP in the saline, MSC, and IL-1β-primed MSC groups by IF (*n* = 6 per group, bar = 50 μm). **G** Expression of CCL2 and TRPV1 in the DRGs of the saline, MSC, and IL-1β-primed MSC groups by IF (*n* = 6 per group, bar = 50 μm). Error bars indicate SD. *p < 0.05, **p < 0.01, ***p < 0.001

**Fig. 8:**
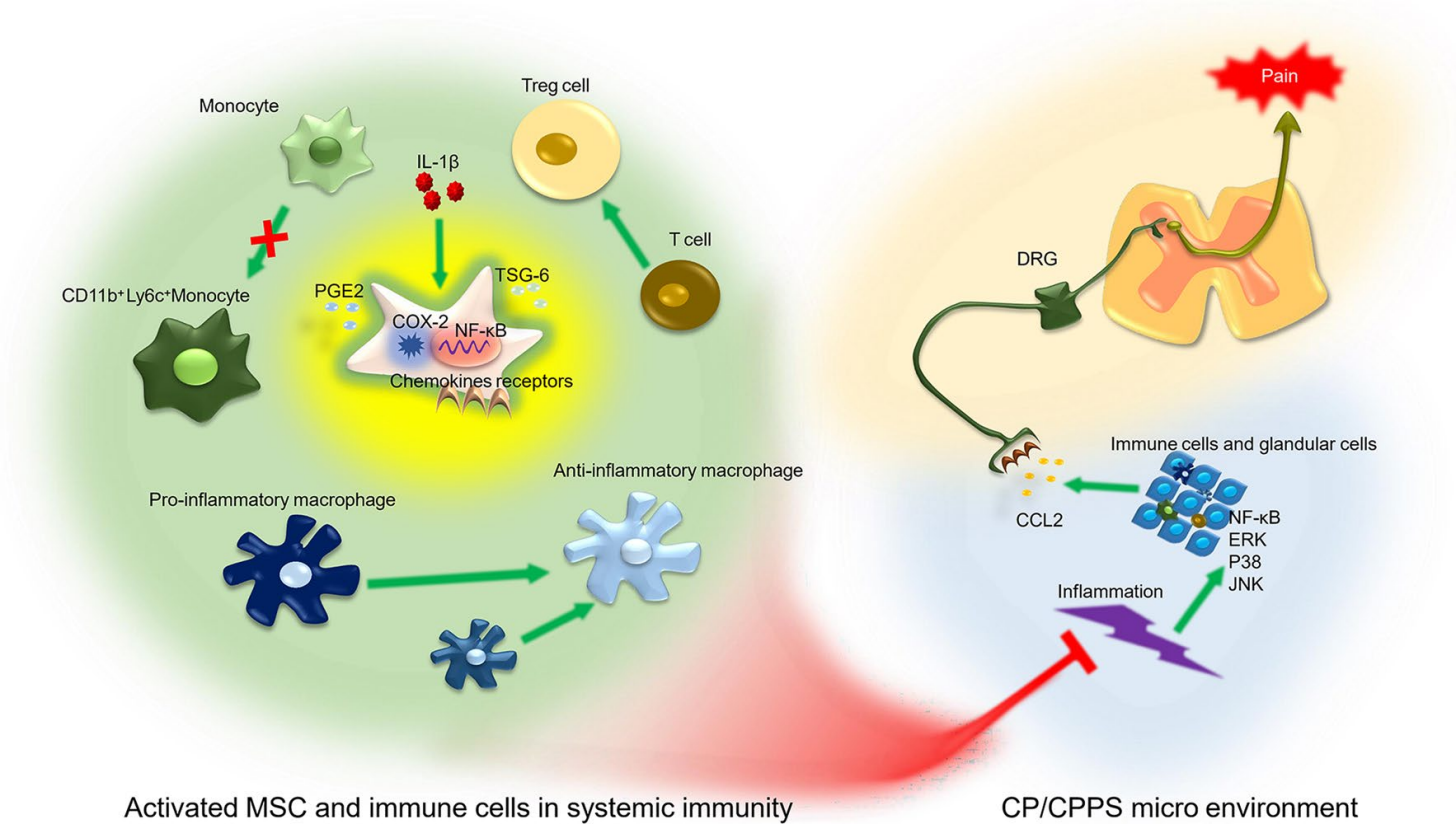
Schematic of IL-1β-primed MSCs in the modulation of macrophages, monocytes, Treg cells, systemic inflammatory response, prostate inflammation, and pelvic pain in CP/CPPS mice. In systemic immunity, IL-1β-primed MSCs regulated the inflammatory properties of macrophages from M1 to M2, CD11b^+^Ly6C^high^ monocyte less infiltration and Treg cell accumulation. The above immune cells enhanced inflammatory resolution, inhibited NF-κB, STAT3 and JNK activation of inflammatory sites and inhibited the release of CCL2 in the prostate via systemic immunity

Chemokines, such as CCL2 and CCL3, can increase the influx of Ca^2+^ ions in DRG receptors, which is a critical mechanism leading to chronic inflammatory pain [58, 59]. Activation of the NF-κB and JNK/MAPK pathways leads to the overexpression of CCL2 [58, 60, 61]. As shown in Fig. 6, the above pathways were downregulated. In CP/CPPS mice, we measured the mRNA expression of the inflammatory chemokines CCL1, 2, 3, 4, 5, 7, 8, 13, 17, and 22. It was found that the expression of CCL2 mRNA in EAP mice was higher than that in control mice by RT-qPCR (Fig. 7A). The increase in CCL2 led to the activation of DRG by increasing Ca^2+^ influx. Through Ca^2+^ fluorescence, it was found that CCL2 can activate DRGs in a manner similar to capsaicin (Fig. 7B) and represent figures (Additional file 2: Figure S7). After IL-1β-primed MSC infusion, we found that mouse pelvic pain gradually decreased in the saline, MSC, and IL-1β-primed MSC groups through behavioral testing (Fig. 7C). Western blotting and IF showed that the expression of CCL2 in the prostate gradually decreased in the saline, MSC, and IL-1β-primed MSC groups (Fig. 7D, E). Moreover, co-staining immune cell epithelial cell marker epithelial cell adhesion molecule (EpCAM) with CCL2, and co-stained immune cell marker CD11b with CCL2. The results showed that CCL2 is mainly expressed in the interstitium of the prostate and immune cells also infiltrated in the interstitium of the prostate. Epithelial cell marker EpCAM mainly expressed in the gland epithelial cells. Besides, CCL2 and immune cells have a large amount of merge (Additional file 2: Figure S8A, B). These results may prove that immune cells express CCL2 in prostate was significantly affected by engraftment of MSCs. In the IF of co-staining Calcitonin gene-related peptide (CGRP) and transient receptor potential cation channel subfamily V member 1 (TRPV1) overexpression in DRGs can maintain inflammatory pain and the activation of DRGs [62]. After MSC infusion, the expression of CGRP in DRGs gradually decreased (Fig. 7F). The expression of CCL2 and TRPV1 in DRGs gradually decreased (Fig. 8G). As shown in Fig. 6, MSCs and IL-1β-primed MSCs reduced inflammation via the JNK/MAPK pathway. At the same time, the expression of the inflammatory pain chemokine CCL2 (a downstream product of the JNK/MAPK pathway) was reduced, which led to decreased activation of DRG nociceptors and pain relief. The study-related mechanism map is shown in Fig. 8.

## Discussion

According to recent studies, MSCs have already been applied in inflammation-related diseases, so MSC infusion may become a new therapeutic strategy for CP/CPPS. Based on the limited immunomodulatory ability and homing efficiency of MSCs, we further used intravenous infusion of IL-1β-primed MSCs for CP/CPPS. We found that its immunoregulatory and MSC-targeted engraftment efficiency on the systemic immune response and local lesions was enhanced by cytokines in CP/CPPS mice.

Currently, treatment options are available for CP/CPPS. In particular, drug therapy has significantly improved over the past decades [63]. At present, NSAIDs and corticosteroids, as the most acceptable therapy choices, could exert anti-inflammatory and symptom-relieving effects on CP/CPPS. However, most of these treatment options focus on the symptoms rather than etiology, and the therapeutic effect is still controversial [64]. Furthermore, these treatments inhibit immunoregulatory ability in all immune cells. This does not allow immune cells to play an immunoregulatory role to resolve inflammation. Therefore, novel strategies to address the disease are still needed. MSCs have emerged as a new therapeutic strategy in many inflammation-related diseases. The immunoregulatory ability of immune cells to restore immunological homeostasis has been widely studied by researchers [65]. Infused MSCs secrete immunoregulatory proteins, such as TSG-6, indoleamine 2,3-dioxygenase (IDO), and PGE-2, to regulate immune cell properties and deployment in the body [66–69]. However, the immunoregulatory ability and homing efficiency of MSCs are still limited [70]. Researchers have begun to find new ways to enhance the therapeutic effects of MSCs. For example, genetically engineered MSCs and cytokine-primed MSCs could endow MSCs with more meaningful therapeutic effects [13, 42, 71]. When MSCs are stimulated by cytokines, the NF-κB pathway in MSCs is activated. Accordingly, their downstream immunomodulatory proteins and chemokine receptors will be overexpressed [42, 47, 72–74]. This means that the targeted migration response to the chemokine capacity of MSCs is strengthened and the MSC homing efficiency is enhanced after cytokine priming [47, 74, 75]. These factors can enhance the immunomodulatory capacities of immune cells and restore the inflammatory microenvironment [72, 74, 76]. The expression of cytokines, such as IL-1β, TNF-α, and MIF, are boosted in inflammatory lesions and the body and recruit a large number of immune cells to maintain the inflammatory microenvironment [77, 78]. Among them, IL-1β is widely increased in CP/CPPS patients and animal models; IL-1β is not only an important inflammatory factor to maintain prostatitis but also one of the main reasons for the maintenance of tissue inflammatory immune cell infiltration [77–79]. Because of its wide existence in inflammatory environments, it was our priming choice. In this study, the immunoregulatory ability of IL-1β-primed MSCs to regulate the inflammatory properties of macrophages and promote the accumulation of Treg cells was enhanced in vitro. IL-1β-primed MSC immunoregulation by regulating the inflammatory properties of macrophages, reducing the deployment of CD11b^+^Ly6C^high^ monocytes and promoting the accumulation of Treg cells in the prostate.

As the largest peripheral immune organ of the body, the spleen is the main filter for blood-borne pathogens and antigens and stores a large population of monocytes and Treg cells [80, 81]. These immune cells can affect the progression of inflammation and migrate among the spleen, bloodstream and local tissues in the body [51, 52]. Under physiological conditions, CD11b + monocytes patrol the spleen, bloodstream and local tissue. CD11b^+^Ly6C^high^ monocytes are inflammatory and migrate more often in inflammatory sites. Conversely, CD11b^+^Ly6C^low^ monocytes can play a role in the resolution of inflammation [82]. Treg cells also exert therapeutic effects on inflammation-related diseases via the resolution of inflammation [81]. The spleen is essential for systemic immunity, which intensively affects the local microenvironment. Proinflammatory immune cells and anti-inflammatory immune cells in the spleen, bloodstream, and prostate are in dynamic equilibrium [83]. When inflammation occurs or inflammation improves, these immune cells can migrate to the local area to achieve proinflammatory or anti-inflammatory effects [82]. After the pretreatment of MSCs with IL-1β, this study found that CXCR4 receptor expression in MSCs increased, and the targeted migration ability in response to CXCL12 was enhanced. However, IL-1β-primed MSCs or MSCs failed to migrate to the prostate. The existence of the blood-prostate barrier may be the reason why CP/CPPS is difficult to treat. The blood–brain barrier-specific antigen EBA is also expressed in the prostate [84, 85]. The prostate and blood vessels have a barrier similar to the blood–brain and blood-testis barriers [85, 86]. Because capillary endothelial cells have special structures and functions, the final concentration of drugs and other therapeutic substances is lower in local tissue than in the bloodstream [87]. Therefore, this study restored the inflammatory microenvironment in the spleen, bloodstream, lung, and prostate through the regulation of the systemic immune response via systemic immune cells. In this study, CD11b^+^Ly6C^high^ inflammatory monocytes were increased in the spleen, bloodstream, lung, and prostate in a CP/CPPS systemic inflammatory environment. After IL-1β-primed MSC infusion, CD11b^+^Ly6C^high^ monocytes were obviously decreased, and inflammation was decreased in the spleen, bloodstream, and prostate. At the same time, the accumulation of Treg cells in the spleen, bloodstream, and prostate was significantly increased. We restored the inflammatory microenvironment in the spleen, bloodstream, lung, and prostate via IL-1β-primed MSC infusion and engraftment to the spleen.

The inflammatory properties of macrophages are critical for restoring the inflammatory microenvironment in the prostate. In tissue, anti-inflammatory and proinflammatory macrophages are in dynamic equilibrium [88]. In inflammatory sites, the inflammatory phenotype of macrophages polarizes to proinflammatory macrophages. This leads to the exacerbation and maintenance of the inflammatory microenvironment. After IL-1β-primed MSC infusion, the proinflammatory phenotype of macrophages polarized to an anti-inflammatory phenotype in lesion tissue [88]. In this study, immune cells restored the inflammatory microenvironment by MSCs regulating the systemic immune response via MSC engraftment in the spleen. This is due to the expression of PGE-2, TSG-6, interleukin 1 receptor antagonist (IL-RN), etc*.* in MSCs, which regulates the inflammatory phenotype of macrophages in remote or adjacent tissues [43, 73, 89, 90]. In this study, immunomodulatory protein genes were overexpressed in IL-1β-primed MSCs. In vitro, IL-1β-primed MSCs regulated the inflammatory phenotype of macrophages. In vivo, infusion of IL-1β-primed MSCs eventually regulated the inflammatory phenotype of macrophages and restored the inflammatory microenvironment in the prostate.

The most unbearable symptom of CP/CPPS patients is chronic pelvic pain. Inflammatory chemokines in the prostate are one of the main causes of chronic pelvic pain [91, 92]. The inflammatory pain-related chemokine CCL2 is also called MCP-1. Related studies have shown that CCL2 expression is increased in CP/CPPS patients and mice [61, 93]. When inflammation occurs in lesions, the overexpression of the NF-κB and JNK/MAPK pathways leads to increased CCL2 secretion [94–98]. It can be used to recruit monocytes/macrophages and bind to DRG C–C motif chemokine receptor (CCR2), leading to the influx of Ca^2+^ in the DRG afferent channel, thereby causing the pain signal to be the same as that of capsaicin [59, 99]. DRGs themselves can also secrete CCL2 to stimulate the nerve conduction pathway of the spinal cord to further cause afferent pain and increase the expression of the TRPV1 nociceptive receptor [100–102]. IL-1β-primed MSC infusion relieved inflammation and regulated the NF-κB and JNK/MAPK pathways, further reducing the expression of CCL2 in local tissues and DRGs. These results proved that MSC therapy can alleviate pelvic pain in CP/CPPS.

In conclusion, we found that the IL-1β-primed MSC immunoregulatory ability and MSC-targeted migration efficiency were enhanced. In addition, it was difficult for IL-1β-primed MSCs to home to the prostate of CP/CPPS mice after MSC infusion. However, we found that IL-1β-primed MSCs engrafted more in the spleen. They regulate local inflammation by reshaping the systemic immune response. Furthermore, the decreased production of the inflammatory chemokine CCL2 through the downregulation of the NF-κB and JNK/MAPK pathways by inflammatory resolution alleviates pain. This may provide a novel therapeutic strategy to explore MSCs with enhanced efficiency for inflammation resolution in CP/CPPS.

## Supplementary Information

**Additional file 1.** Supplemental tables.

**Additional file 2.** Supplemental figures.

## Data Availability

The data and material to reproduce these findings are available from the authors by reasonable request.

## Abbreviations

CP/CPPS: Chronic prostatitis/Chronic pelvic pain syndrome
MSCs: Mesenchymal stromal cells
IL-1β: Interleukin-1β
EAP: Experimental autoimmune prostatitis
CCL2: Chemokine (C–C motif) ligand 2
TSG-6: TNF-stimulated gene 6 protein
PGE-2: Prostaglandin E2
CXCR4: Chemokine C-X-C motif receptor 4
AVMA: American Veterinary Medical Association
NOD-EAP: Non-obesity diabetes-experimental autoimmune prostatitis
MAG: Male reproductive accessory gland
CFA: Complete Freund's adjuvant
DRGs: Dorsal root ganglia
CXCL12: Chemokine (C-X-Cmotif) ligand 12
IF: Immunofluorescence
HE: Hematoxylin–eosin
Arg1: Arginase 1
iNOS: Inducible nitric oxide synthase enzyme
IDO: Indoleamine 2,3-dioxygenase
IL-RN: Interleukin 1 receptor antagonist
EpCAM: Epithelial cell adhesion molecule
CGRP: Calcitonin gene-related peptide
TRPV1: Transient receptor potential cation channel subfamily V member 1
