# Supplementary material for: IL-1β-primed mesenchymal stromal cells exert enhanced therapeutic effects to alleviate Chronic Prostatitis/Chronic Pelvic Pain Syndrome through systemic immunity
Source: Stem Cell Res Ther. 2021 Sep 25;12:514. doi: 10.1186/s13287-021-02579-0 (PMC8466748; doi:10.1186/s13287-021-02579-0)
Supplement: Supplementary file 1 — Additional file 1. Supplemental tables. [file 13287_2021_2579_MOESM1_ESM.docx]

| **Primary antibody:** |  |  |
| --- | --- | --- |
| CD11b/ITGAM Rabbit pAb | 1:100(IF) | ZENBIO(380675) |
| MCP1/CCL2 (5H2) Mouse mAb | 1:1000(WB)  1:200(IF) | ZENBIO(220691) |
| IL1β Rabbit Ab | 1:1000(WB) | Affinity (DF6251) |
| GAPDH Rabbit Ab | 1:3000(WB) | Affinity (AF7021) |
| CXCL12 Rabbit Ab | 1:1000(WB) | Abcam (ab9797) |
| CXCR4 Mouse Ab | 1:1000(WB) | Proteintech (60042-1-Ig) |
| P-STAT3 Rabbit Ab | 1:1000(WB) | CST (9131) |
| STAT3 Mouse Ab | 1:1000(WB) | CST (9139) |
| p-P65 Rabbit Ab | 1:1000(WB) | CST (3031) |
| P65 Rabbit Ab | 1:1000(WB) | CST (8242) |
| p-JNK Rabbit Ab | 1:1000(WB) | CST (4668) |
| JNK Rabbit Ab | 1:1000(WB) | CST (9252) |
| p-P38 Rabbit Ab | 1:1000(WB) | CST (4511) |
| P38 Rabbit Ab | 1:1000(WB) | CST (8690) |
| p-ERK Rabbit Ab | 1:1000(WB) | CST (4370) |
| ERK Rabbit Ab | 1:1000(WB) | CST (4695) |
| CD206 | 1:100(IF) | Proteintech (18704-1-AP) |
| CD86 | 1:100(IF) | Proteintech (13395-1-AP) |
| CGRP Rabbit Ab | 1:100(IF) | Abcam (8198) |
| TRPV1 Rabbit Ab | 1:100(IF) | Alomone Labs (ACC-030) |
| **Secondary antibody:** |  |  |
| Anti-mouse IgG HRP-linked Ab | 1:5000 | CST (7076) |
| Anti-rabbit IgG HRP-linked Ab | 1:5000 | CST (7074) |
| Anti-rabbit IgG (Fluor® 488 Conjugate) | 1:500 | CST (4412) |
| Anti-mouse IgG (Fluor® 488 Conjugate) | 1:500 | CST (4408) |
| **Antibodies used in flow cytometry** |  |  |
| CD11b-PE/CY7 Antibody | 1:100 | BioLegend (101215) |
| Ly6C-APC Antibody | 1:100 | BioLegend (128015) |
| CD45-PE/CY7 Antibody | 1:100 | BioLegend (103113) |
| F4/80-BV421 Antibody | 1:100 | BioLegend (123137) |
| CD86-PE Antibody | 1:100 | BioLegend (105105) |
| CD206-APC Antibody | 1:100 | BioLegend (141707) |
| INOS-PE Antibody | 1:100 | Invitrogen (12-5920-80) |
| Arg1-APC Antibody | 1:100 | Invitrogen (17-3697-82) |
| CD4-PE Antibody | 1:100 | BioLegend (100407) |
| Foxp3-APC Antibody | 1:100 | BioLegend (320113) |

Table S1: Primary and secondary antibodies used for immunostaining, Western blotting, or flow cytometry.

| GENE | PRIMER SEQUENCE (5'-3'） | |
| --- | --- | --- |
| CCL2 | CAGCCAGATGCAATCAATGCC |  |
| CCL7  CCL12 | TGGAATCCTGAACCCACTTCT  GCTGCTTTCAGCATCCAAGTG  CCAGGGACACCGACTACTG  ATTTCCACACTTCTATGCCTCCT  ATCCAGTATGGTCCTGAAGATCA | |
| CCL8 | TGCTGAAGCTCACACCCTTG | |
|  | GGAATGGAAACTGAATCTGGCTG | |
| CXCL3 | TGCATCAGTGACGGTAAACCA | |
|  | TTCTTCAGCCGTGCAACAATC | |
| IL-23 | ATGCTGGATTGCAGAGCAGTA | |
|  | ACGGGGCACATTATTTTTAGTCT | |
| IL-1α | GCACCTTACACCTACCAGAGT | |
|  | AAACTTCTGCCTGACGAGCTT | |
| IFN-α | ATGAACGCTACACACTGCATC | |
|  | CCATCCTTTTGCCAGTTCCTC | |
| TNF-α | CCCTCACACTCAGATCATCTTCT | |
|  | GCTACGACGTGGGCTACAG | |
| IL-12 | TGGTTTGCCATCGTTTTGCTG | |
|  | ACAGGTGAGGTTCACTGTTTCT | |
| IL-1β | GCAACTGTTCCTGAACTCAACT | |
|  | ATCTTTTGGGGTCCGTCAACT | |
| IL-10 | GCTCTTACTGACTGGCATGAG | |
|  | CGCAGCTCTAGGAGCATGTG | |
| IL-6 | TAGTCCTTCCTACCCCAATTTCC | |
|  | TTGGTCCTTAGCCACTCCTTC | |
| IL-27 | CTGTTGCTGCTACCCTTGCTT | |
|  | CACTCCTGGCAATCGAGATTC | |
| IL-17 | TTTAACTCCCTTGGCGCAAAA | |
|  | CTTTCCCTCCGCATTGACAC | |
| IFN-β | CAGCTCCAAGAAAGGACGAAC | |
|  | GGCAGTGTAACTCTTCTGCAT | |
| GM-CSF | GGCCTTGGAAGCATGTAGAGG | |
|  | GGAGAACTCGTTAGAGACGACTT | |
| MMP2 | CAAGTTCCCCGGCGATGTC | |
|  | TTCTGGTCAAGGTCACCTGTC | |
| MMP9 | CTGGACAGCCAGACACTAAAG | |
|  | CTCGCGGCAAGTCTTCAGAG | |
| MMP12 | GAGTCCAGCCACCAACATTAC | |
|  | GCGAAGTGGGTCAAAGACAG | |
| MMP13 | CTTCTTCTTGTTGAGCTGGACTC | |
|  | CTGTGGAGGTCACTGTAGACT | |
| iNOS | GTTCTCAGCCCAACAATACAAGA | |
|  | GTGGACGGGTCGATGTCAC | |
| CD86 | TGTTTCCGTGGAGACGCAAG | |
|  | TTGAGCCTTTGTAAATGGGCA | |
| Chil3 | CAGGTCTGGCAATTCTTCTGAA | |
|  | GTCTTGCTCATGTGTGTAAGTGA | |
| Retnlg | CTTGCCAATCGAGATGACTGT | |
|  | ACCCAGTAGCAGTCATCCCA | |
| Mrc1 | CTCTGTTCAGCTATTGGACGC | |
|  | CGGAATTTCTGGGATTCAGCTTC | |
| IL-4 | GGTCTCAACCCCCAGCTAGT | |
|  | GCCGATGATCTCTCTCAAGTGAT | |
| CCL3 | TTCTCTGTACCATGACACTCTGC | |
|  | CGTGGAATCTTCCGGCTGTAG | |
| CCL4 | TTCCTGCTGTTTCTCTTACACCT | |
|  | CTGTCTGCCTCTTTTGGTCAG | |
| CCL5 | GCTGCTTTGCCTACCTCTCC | |
|  | TCGAGTGACAAACACGACTGC | |
| CXCL2 | TGTGACGGCAGGGAAATGTA | |
|  | TGCTCTAACACAGAGGGAAACA | |
| CXCL10 | CCAAGTGCTGCCGTCATTTTC | |
|  | GGCTCGCAGGGATGATTTCAA | |
| CXCL12 | TGCATCAGTGACGGTAAACCA | |
|  | TTCTTCAGCCGTGCAACAATC | |
| GAPDH | AGGTCGGTGTGAACGGATTTG | |
|  | TGTAGACCATGTAGTTGAGGTCA | |
| CCR1 | CCTGCTGACGATTGACAGGTA  TCTCGTAGGCTTTCGTGAGGA | |
| CCR2 | TACGGTGCTCCCTGTCATAAA  TAAGATGAGGACGACCAGCAT | |
| CCR3 | TGGCATGTGTAAGCTCCTCTC  CCTGTCGATTGTCAGCAGGATTA | |
| CCR4 | AGAAGGCATCAAGGCATTTGG  ACACATCAGTCATGGACCTGAG | |
| CCR5 | GTTGGACCAAGCTATGCAGGT  GCAGAAGCGTTTGGCAATGT | |
| CCR6 | GGCTATTTGTACCGATTGCCT  GATGCCTTTTAGCAACTTGCAC | |
| CXCR1 | GATGCCTTTTAGCAACTTGCAC  CCAGGACCTCATAGCAAACTG | |
| CXCR2 | CCTGTCTTACTTTTCCGAAGGAC  TTGCTGTATTGTTGCCCATGT | |
| CXCR3 | GGTCATGGCCTACTGCTATGC  CCACGTCTACCCTGCTTTCT | |
| CXCR4 | ACTACACCGAGGAAATGGGCT  CCCACAATGCCAGTTAAGAAGA | |
| CXCR5 | GGTCACCCTACCACATCGTC  GCCATTCAGCTTGCAGGTATTG | |
| CXCR6 | GACTATGGGTTCAGCAGTTTCA  GGCTCTGCAACTTATGGTAGAAG | |
| TSG-6 | CGTCTCGCAACCTACAAGCA  GGTATCCGACTCTACCCTTGG | |
| COX-2 | TTCCAATCCATGTCAAAACCGT  AGTCCGGGTACAGTCACACTT | |
| IL-RN | TAGACATGGTGCCTATTGACCT  TCGTGACTATAAGGGGCTCTTC | |

Table S2: Primers used to amplify transcripts during RT-qPCR analysis.
