# Supplementary material for: IL-1β-primed mesenchymal stromal cells exert enhanced therapeutic effects to alleviate Chronic Prostatitis/Chronic Pelvic Pain Syndrome through systemic immunity
Source: Stem Cell Res Ther. 2021 Sep 25;12:514. doi: 10.1186/s13287-021-02579-0 (PMC8466748; doi:10.1186/s13287-021-02579-0)
Supplement: Supplementary file 2 — Additional file 2. Supplemental figures. [file 13287_2021_2579_MOESM2_ESM.docx]

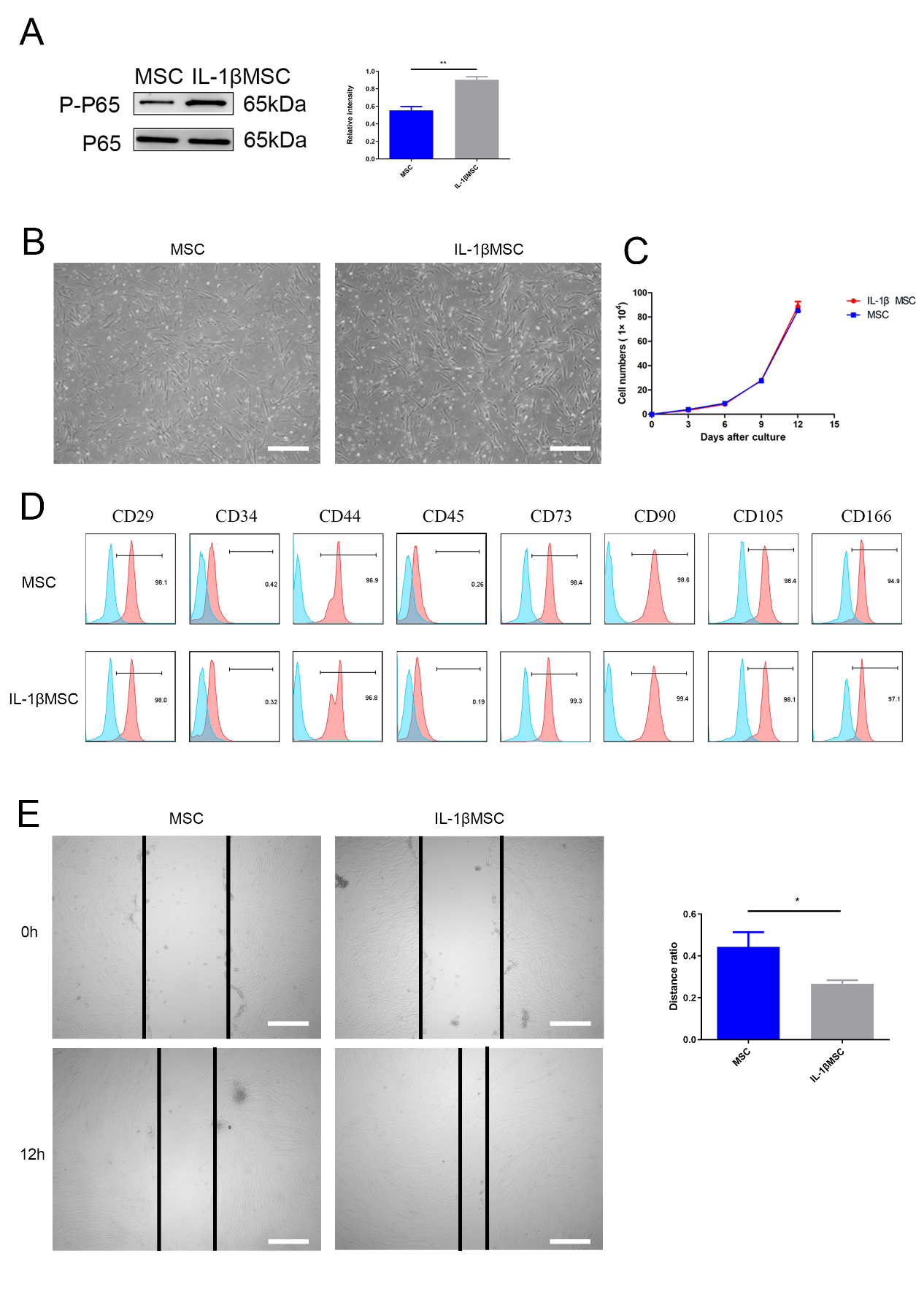


**Figure S1: IL-1β primed MSCs higher expressed NF-κB pathways and did not alter the intrinsic characteristics of MSCs**. A. p-P65 expressed in IL-1β primed MSCs by Western blot. B. Representative images of MSCs and IL-1β primed MSCs under bright field microscopy in passage 5 (Bar = 200μm). C. Proliferation of MSCs and IL-1β primed MSCs in vitro. D. The expression of MSC surface markers in IL-1β primed MSCs and MSCs. E. Scratch test shows migration ability of IL-1β primed MSCs and MSCs (Bar = 500μm).


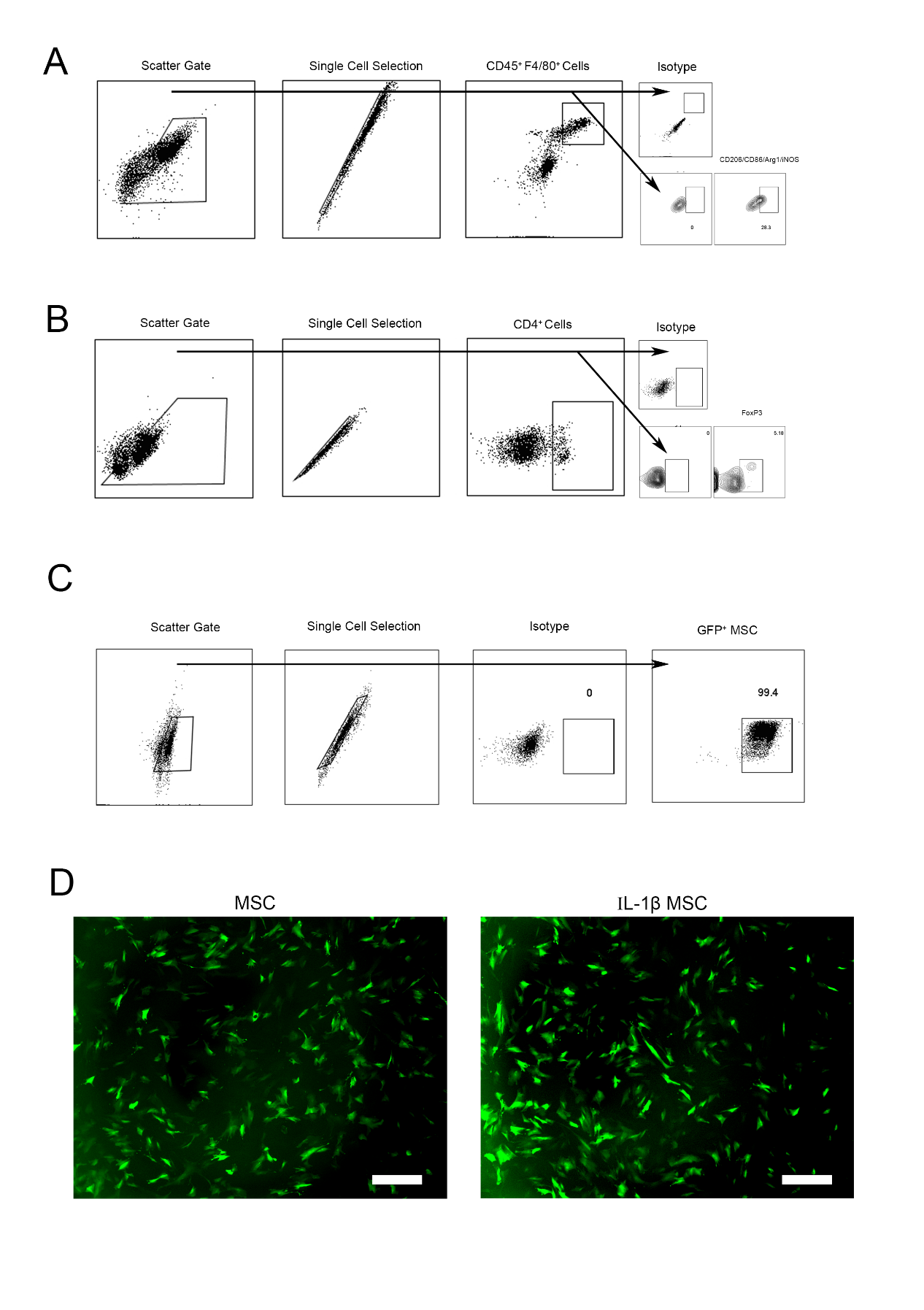


**Figure S2: Gate selection for macrophages and CD4^+^FoxP^+^ Treg cells.** A. Macrophages gate selection. B. Treg cells gate selection.


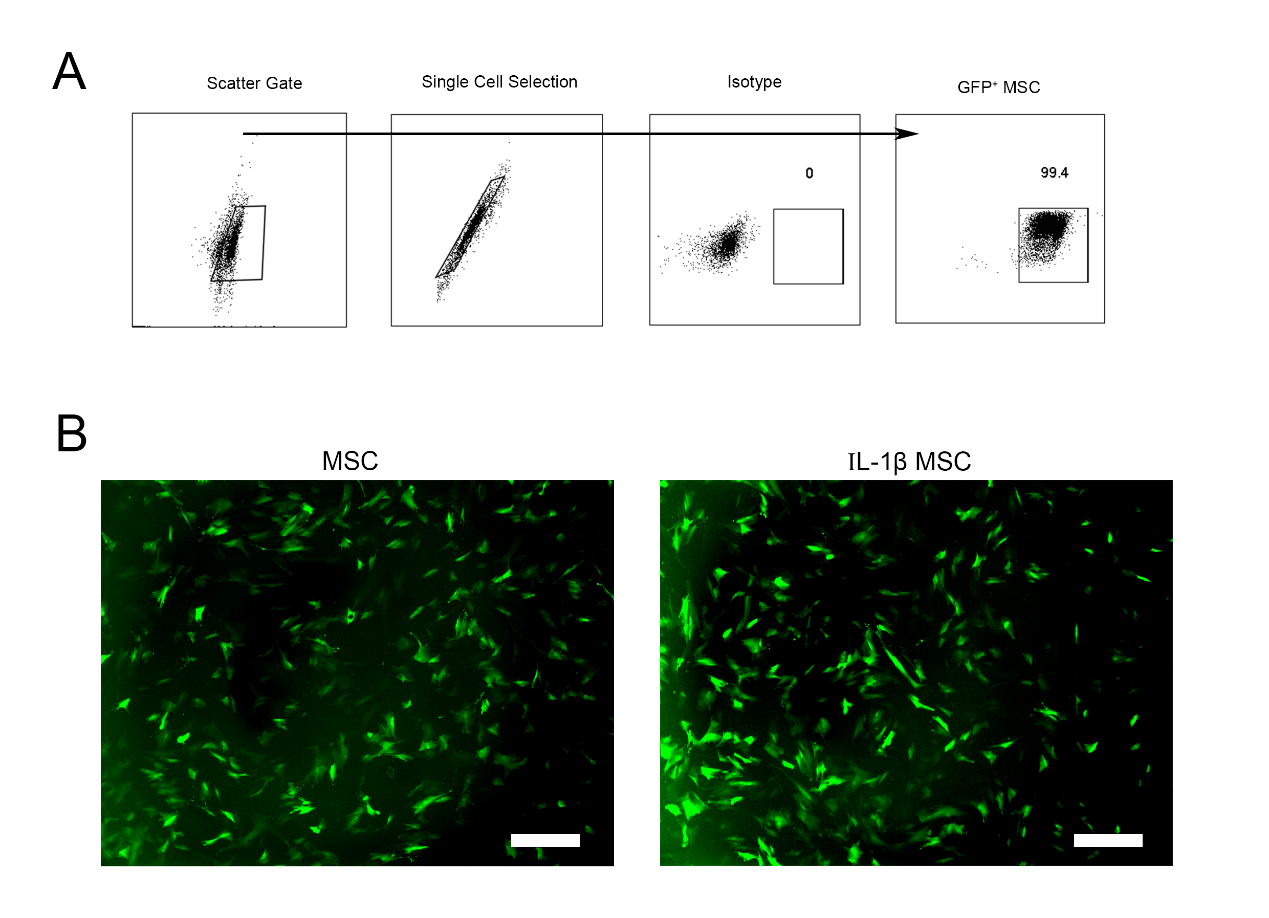


**Figure S3: Acquisition of GFP+MSCs.** A. Flow cytometry sorting GFP^+^ MSCs cells. B. Observe two kinds of cells under the IF microscope (Bar = 200μm).


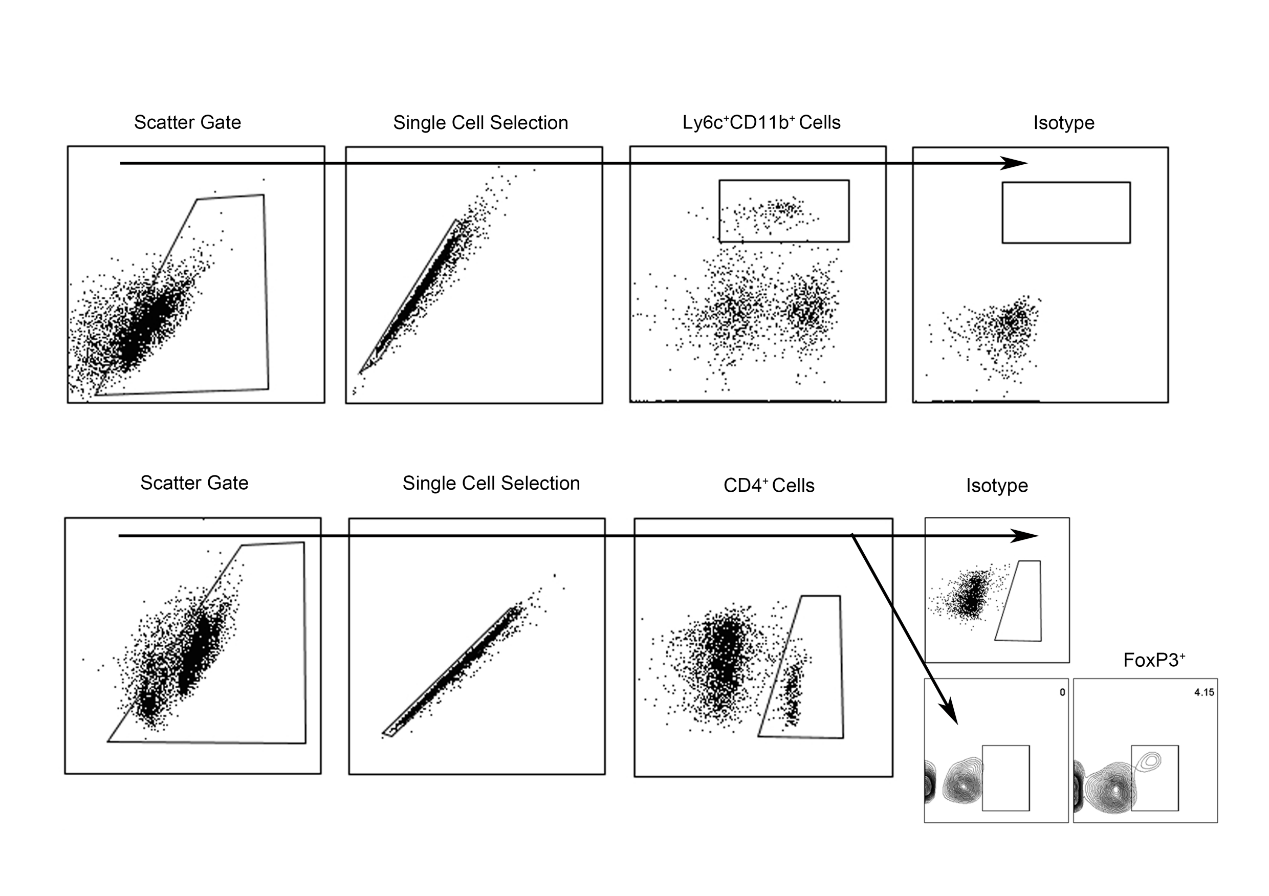


**Figure S4:** Gate selection of macrophages and CD4^+^FoxP^+^ Treg cells in spleen, bloodstream, lung and prostate.

**

**

**Figure S5:** Selection of in situ macrophages in EAP mice prostate.


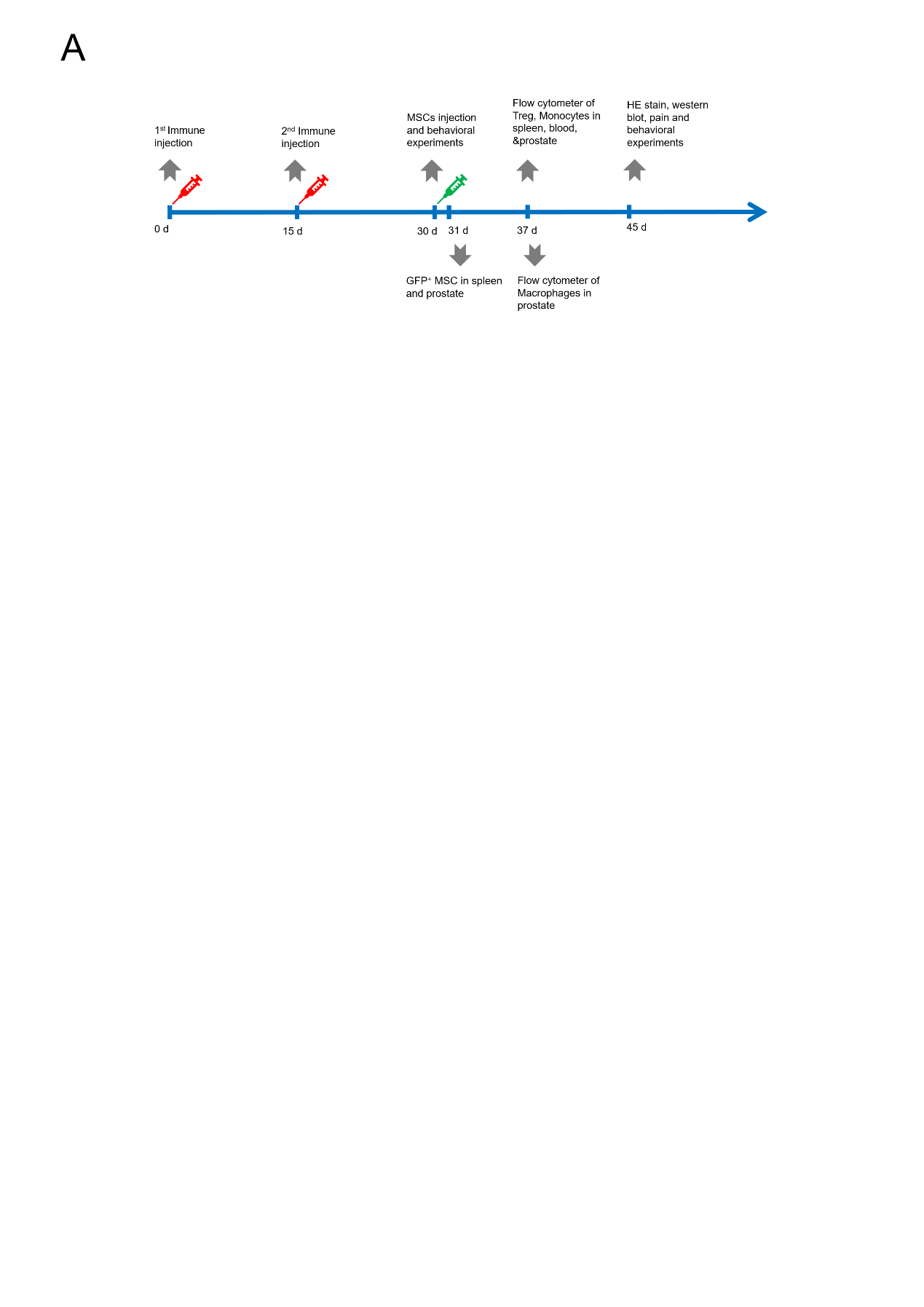


**Figure S6: Schematic of the experimental procedures.** At 30^th^ day, pelvic pain was checked by Von Frey test. GFP^+^MSCs and GFP^+^ IL-1β MSCs were injected into EAP mice. At 31^th^ day, spleen and prostate of EAP mice after GFP^+^MSCs infusion were collected to analyze the MSCs engraftment in spleen and prostate. At 37^th^ day, spleen, blood and prostate were collected from MSCs infusion EAP mice for flow cytometry, IF. At 45^th^ day, pelvic pain was checked by Von Frey test. DRGs and prostate were collected from MSCs infusion EAP mice for flow cytometry, IF and Western blot.


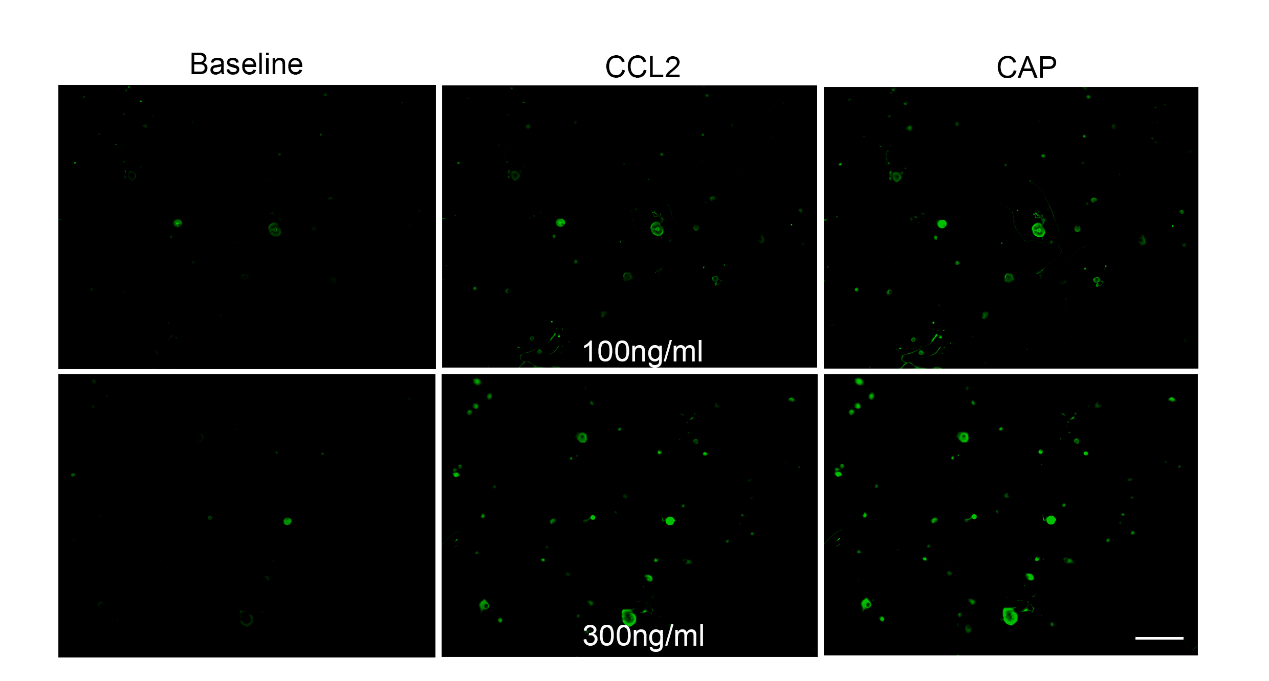


**Figure S7:** Representative images of calcium responses to CCL2 and capsaicin (Bar=100μm).


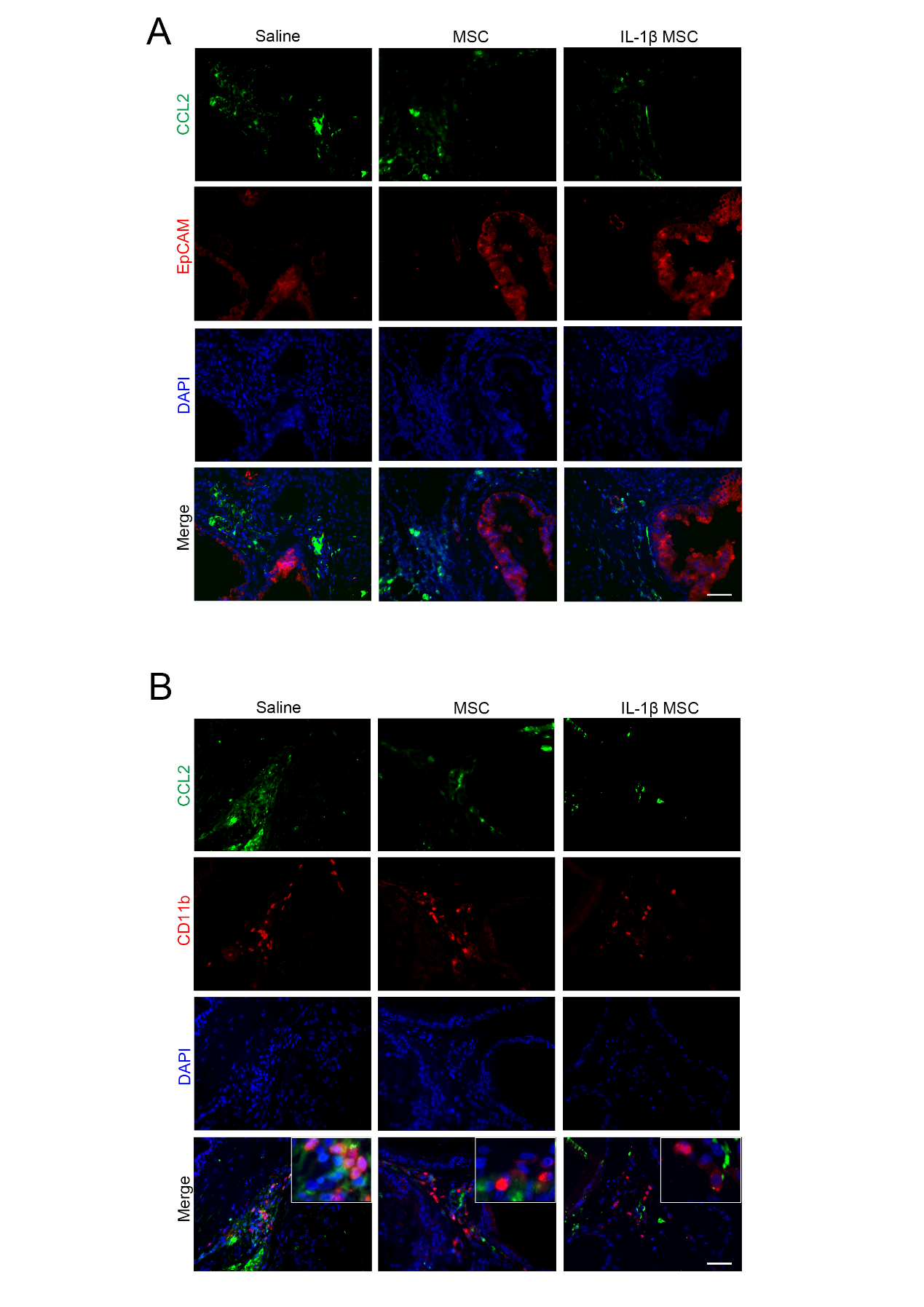


**Figure S8: Representative images suggested CCL2 was mainly secreted by immune cells infiltrating the interstitium of the prostate.** A. Co-staining of epithelial cells marker EpCAM and CCL2. B. Co-staining of immune cells marker CD11b and CCL2 (Bar = 50μm).
